# Translational Analysis of Moderate to Severe Asthma GWAS Signals Into Candidate Causal Genes and Their Functional, Tissue-Dependent and Disease-Related Associations

**DOI:** 10.3389/falgy.2021.738741

**Published:** 2021-10-18

**Authors:** Michael A. Portelli, Kamini Rakkar, Sile Hu, Yike Guo, Ian M. Adcock, Ian Sayers

**Affiliations:** ^1^Centre for Respiratory Research, Translational Medical Sciences, School of Medicine, National Institute for Health Research Nottingham Biomedical Research Centre, Nottingham University Biodiscovery Institute, University of Nottingham, Nottingham, United Kingdom; ^2^Data Science Institute, Imperial College London, London, United Kingdom; ^3^The National Heart and Lung Institute, Imperial College London, London, United Kingdom

**Keywords:** GWAS, causal genes, eQTL, SNP, moderate to severe asthma

## Abstract

Asthma affects more than 300 million people globally and is both under diagnosed and under treated. The most recent and largest genome-wide association study investigating moderate to severe asthma to date was carried out in 2019 and identified 25 independent signals. However, as new and in-depth downstream databases become available, the translational analysis of these signals into target genes and pathways is timely. In this study, unique (U-BIOPRED) and publicly available datasets (HaploReg, Open Target Genetics and GTEx) were investigated for the 25 GWAS signals to identify 37 candidate causal genes. Additional traits associated with these signals were identified through PheWAS using the UK Biobank resource, with asthma and eosinophilic traits amongst the strongest associated. Gene expression omnibus dataset examination identified 13 candidate genes with altered expression profiles in the airways and blood of asthmatic subjects, including *MUC5AC* and *STAT6*. Gene expression analysis through publicly available datasets highlighted lung tissue cell specific expression, with both *MUC5AC* and *SLC22A4* genes showing enriched expression in ciliated cells. Gene enrichment pathway and interaction analysis highlighted the dominance of the *HLA-DQA1/A2/B1/B2* gene cluster across many immunological diseases including asthma, type I diabetes, and rheumatoid arthritis. Interaction and prediction analyses found *IL33* and *IL18R1* to be key co-localization partners for other genes, predicted that *CD274* forms co-expression relationships with 13 other genes, including the *HLA-DQA1/A2/B1/B2* gene cluster and that *MUC5AC* and *IL37* are co-expressed. Drug interaction analysis revealed that 11 of the candidate genes have an interaction with available therapeutics. This study provides significant insight into these GWAS signals in the context of cell expression, function, and disease relationship with the view of informing future research and drug development efforts for moderate-severe asthma.

## Introduction

Asthma is one the of most predominant non-communicable diseases throughout the world. It accounts for over 400,000 deaths per year and by World Health Organization estimates, 24.8 million disability adjusted life years (DALYS) were attributable to Asthma in 2016 (1). Although there is no cure for asthma, most symptoms can be managed well with medication. However, patients with severe asthma, which represent ~4% of all patients, suffer from uncontrolled symptoms and frequent exacerbations despite medication (2).

Over the years, many genome and phenome wide association studies (GWAS, PheWAS) have been completed resulting in a large number of signals mainly *via* single nucleotide polymorphisms (SNPs) associated with asthma relevant traits (3–7), which have been reviewed in detail (8, 9). GWAS and PheWAS (10–12) have greatly advanced asthma research and translating these signals into candidate causal genes is the next step in moving to greater mechanistic understanding of asthma, therapeutic targets and pathways for investigation. However, many single SNPs are largely defined and generally mapped to the closest gene, regardless of whether the SNP has any effect on the function of that gene e.g., expression. This ignores the complexity of the 3D architecture of DNA which may result in SNPs linearly far away from a gene being closer than thought in the 3D structure and having a functional role (13). Indeed, it has been shown that over 90% of disease-linked variants are located in non-coding regions of the genome (14). Therefore, accurately determining causal genes is important for understanding the biology underlying GWAS and PheWAS signals, especially within respiratory relevant tissues and compartments, including the lung. An additional layer of complexity is the possibility of specific SNP-tissue interactions, where SNP-gene regulation may occur differently in different tissues and environments (15, 16).

In this study, we investigated the 25 signals (including four novel signals; rs11603634, rs1090584, rs10905284, and rs61816761) identified in the recent Moderate to Severe Asthma GWAS (7). We aimed to identify candidate causal genes from these signals and understand their association with asthma, highlighting potential targets for downstream investigation utilizing unique [Unbiased biomarkers for the prediction of respiratory disease outcomes (U-BIOPRED)] and publicly available datasets. We then investigated these genes *via* multiple gene enrichment and interaction analyses and gene expression analysis in the lung and in whole blood. This study represents a significant advance in our understanding of the mechanistic underpinnings of these signals and provides candidate genes and pathways for future drug development in moderate-severe asthma.

## Materials and Methods

### Signal to Trait Association Analysis—PheWAS Analysis From GeneATLAS UK Biobank Data

**Figure 1:**
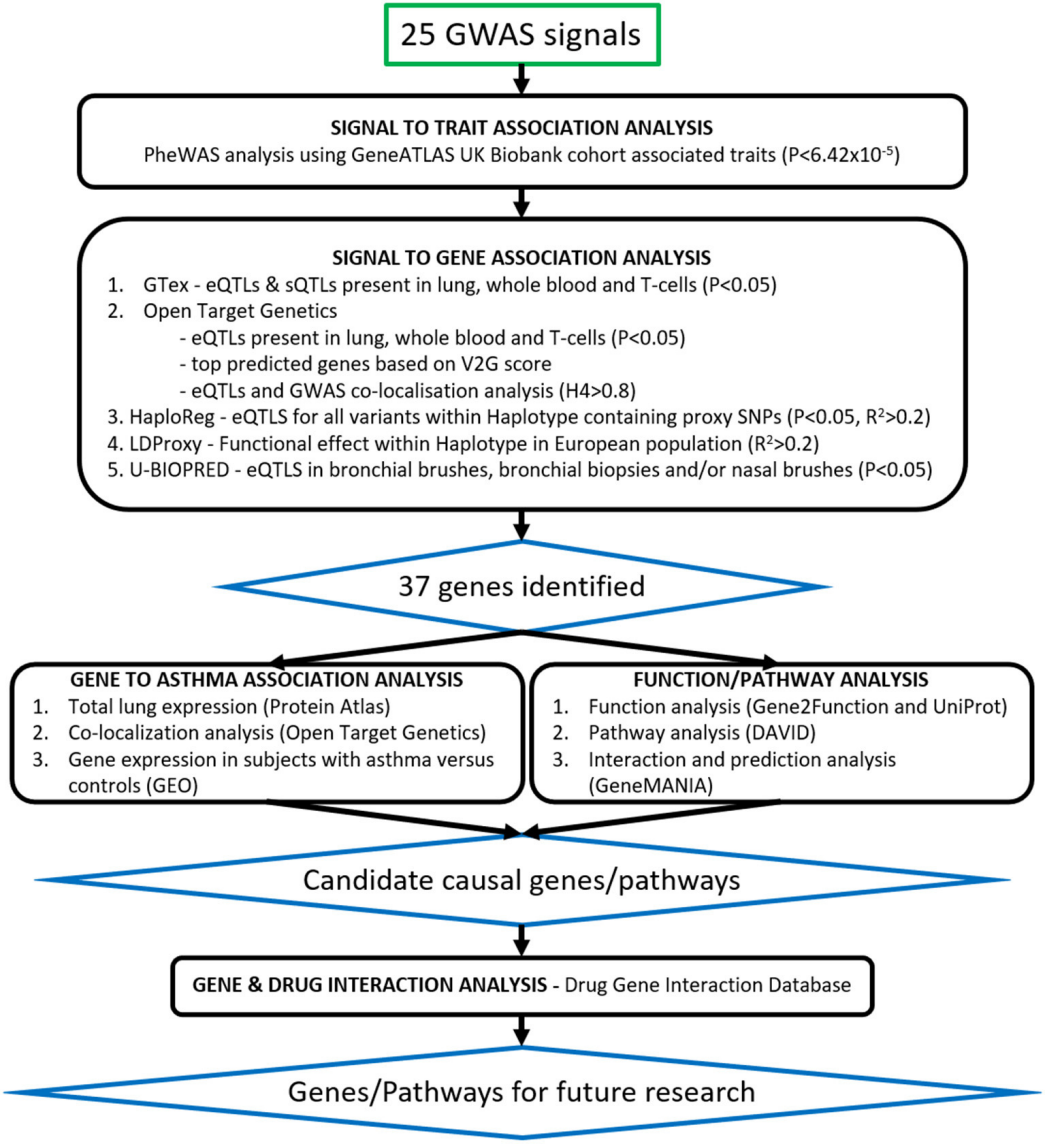
Translational analysis pipeline. Twenty-five signals from the Moderate to Severe Asthma GWAS were taken through signal to trait and signal to gene association analyses. Signals were searched for in publicly available databases such as GeneATLAS, PheWAS, Open Target Genetics, GTex, HaploReg and LDLink as well as our U-BIOPRED (UB) datasets of Bronchial Biopsies, Bronchial Brushes, and Nasal Brushes. Gene association was scored as per Table 3 and 37 genes were identified. Candidate causal genes were then searched for in gene expression datasets from subjects with asthma vs. controls and single-cell-RNA lung tissue datasets. Asthma related pathways and interactions between genes were identified using the DAVID Functional Annotation Tool and GeneMANIA prediction server and drug gene interactions were identified using the Drug Gene Interaction Database.

To investigate traits associated with the signals, 25 signals taken from the Moderate to Severe Asthma GWAS were individually searched for in the online GeneATLAS database (17) according to the risk allele. This database of associations between traits and variants uses the UK Biobank cohort (18). It has 778 traits and therefore with Bonferroni correction an adjusted *P*-value of 6.42 × 10^−5^ was used to determine significance. Proxies were used for the following signals which were not present in the database: rs61816761 (rs61816766, *R*^2^ = 0.50), rs367983479 (rs1504215, *R*^2^ = 0.85), rs71266076 (rs7824993, *R*^2^ = 0.81), rs7305461 (rs1131017, *R*^2^ = 0.75), rs112502960 (rs62076439, *R*^2^ = 1.0), rs61840192 (rs1031163, *R*^2^ = 1.0), rs560026225 (rs72687036, *R*^2^ = 0.66), and rs776111176 (rs3997872, *R*^2^ = 0.82). Displayed traits were selected and broadly grouped into asthma, blood/immune cell, allergic, other respiratory, other inflammatory, and auto-immune categories. An overview of the project pipeline is provided in Figure 1.

### Signal to Gene Association Analysis

To determine likely signal to gene associations in lung tissue, T-cells and whole blood, we interrogated publicly available databases (GTEx V8, Open Target Genetics (OTG), HaploReg V4.1, LD Matrix 5.0), a unique dataset presenting expression quantitative trait loci (eQTLs) for bronchial epithelial brushes (*n* = 117), bronchial biopsies (*n* = 84), and nasal epithelial brushes (*n* = 75) (U-BIOPRED), as well as a general literature search for previously published associations. Using these datasets, we noted eQTLs [GTEx, OTG (19), HaploReg (20), U-BIOPRED], sQTLs (GTEx), variant 2 gene scores (OTG), Gene to eQTL localization (OTG), and tagged functional variation [LDLink (LDProxy) (21)] for the sentinel SNP reported for these signals in the original study or, in cases where the sentinel SNP was not covered, a proxy SNP. We used a corrected significance threshold of *P* < 0.05, as presented in each dataset, to identify eQTLs across all datasets, and a H4 > 0.8 threshold for the Gene to eQTL co-localization. Proxy SNPs were selected based on linkage disequilibrium *R*^2^ scores from the publicly available reference haplotypes for the EUR super population (CEU+GBR+TSI+FIN+IBS) from 1000_Phase 3 (Version 5) of the 1,000 Genomes Project accessed through LDLink, where the selected proxy was the SNP with the highest *R*^2^ value (minimum *R*^2^ = 0.2) that was present in the interrogated database.

Each signal to gene association identified was given a score point, with greater weighting given to U-BIOPRED eQTLs due to the specificity of the eQTLs to the respiratory related samples. This generated a total score, where genes scoring s ≥ 3 were selected for further downstream analysis. In cases where no genes achieved s ≥ 3 for a particular signal, the gene with the highest available score was selected. In the case of signal rs61816761, where all candidate genes scored below 3, *FLG* was selected as the most likely candidate causal gene due to existing literature (22, 23).

### Gene Function and Disease Association Analysis

Gene function was determined using the Gene 2 Function (24) and UniProt (25) databases, and gene function and biological processes annotations were listed.

### Total and Single Cell RNA Expression Analysis in Lung Tissue

To determine the candidate gene expression profile in lung tissue, the Human Protein Atlas (HPA, http://www.proteinatlas.org) resource was used (26). For total lung expression, normalized expression (NX) values were used. The HPA provides NX values through normalizing the average transcript per million (TPM) value of all individual lung samples from three transcriptomics datasets (HPA, GTEx, and FANTOM5). For single cell (sc) lung tissue gene expression, transcripts per million protein coding genes (pTPM) values were used. The HPA provides pTPM values through normalization of read counts from four lung tissue datasets (Single Cell Expression Atlas, the Human Cell Atlas, the Gene Expression Omnibus (GEO) and the European Genome-phenome Archive). Both NX and pTPM values were obtained for 36 candidate genes (*FLG* had no data available). log_10_(pTPM) values were used for the heatmap. Genes which showed enriched/enhanced expression for lung epithelial (blue bars) or blood/immune cells (red bars) were highlighted in the bar chart. Enriched/enhanced expression was determined by using the same scoring system as the HPA. Enriched/enhanced expression is defined by the HPA as having NX values at least four times of any other tissue/cell type from their full transcriptomics data of 37 tissues and 43 single cell types.

### Co-localization Analysis of Tissue Gene Expression and the Reported Trait “Asthma”

To identify evidence linking gene expression across tissue/cell types with the asthma trait, the OTG portal was used. H4 scores for studies where asthma was the exclusive trait were obtained for the 37 candidate genes focusing on lung tissue and/or blood/immune cell types. Median H4 scores of all variants (signals) were plotted for each gene. Genes *AAGAB, CD247, DEXI, FLG, GATA3, HLA-DQA1/A2, B2, IL33, KIF1A, KIAA1109, MSL1, MUC5AC, PGAP3*, and *ZBTB10* had no association data for lung/blood/immune cells/tissue and therefore have not been included in the analysis.

### Differential mRNA Expression Analyses in Airway Epithelial Cells Isolated From Subjects With Asthma or Control Subjects

Genes which showed enriched epithelial expression in the HPA database and/or colocalization in lung tissue (median H4 > 0.8) were selected for further investigation to explore mRNA expression in bronchial epithelial cells isolated from subjects with asthma or control subjects. The freely available U-BIOPRED gene expression dataset, GSE43696, was used from the GEO resource (27), as it was the largest adult dataset available with a control group. The GSE43696 dataset comprises of Agilent Human GE 4×44K V2 Gene Expression data of bronchial epithelial cells for 20 control, 50 mild-moderate, and 38 severe asthma subjects (28, 29). In this dataset, mild-moderate asthma was defined as subjects having an FEV_1_ of >60% predicted, with/without low-moderate dose inhaled corticosteroids. Severe asthma was defined as subjects having severe refractory asthma, including the continuous use of high-dose inhaled corticosteroids and/or frequent use of oral corticosteroids with continuing symptoms and/or chronic airflow limitation. Control subjects had normal lung function, no history of respiratory disease or recent respiratory infection and no evidence of bronchial hyperresponsiveness. Expression values were plotted for each gene stratified by subject group. Data was statistically analyzed using a Kruskal-Wallis test with a two-stage linear step-up procedure of Benjamini, Krieger, and Yekutieli to control the FDR at 5%. *P* < 0.05 was considered significant.

### Differential mRNA Expression Analyses in Blood Cells Isolated From Subjects With Asthma or Control Subjects

Candidate genes which showed enriched blood cell expression in the HPA database or colocalization in blood (median H4 > 0.8) were selected for further investigation to explore mRNA expression in blood cells between asthma and control subjects. The freely available gene expression dataset with adult subjects which also included a control group, GSE69683, was used from the GEO resource (27). The GSE69683 dataset comprises of Affymetrix HT HG-U133+ PM Array gene expression data of blood cell samples with 87 control, 77 mild-moderate, and 246 severe asthma subjects from the U-BIOPRED cohort (30). Samples were selected based on their non-smoker status (<5 pack-year smoking history). Mild-moderate asthma was defined as having controlled or partially controlled asthma symptoms, as defined by the Global Initiative for Asthma (GINA), whilst receiving a dose of <500μg fluticasone propionate/day or equivalent. Severe asthma was defined as having uncontrolled symptoms defined according to GINA guidelines and/or frequent exacerbations (more than two per year) despite high-dose inhaled corticosteroids (ICS) (ICS ≥ 1,000 μg fluticasone propionate/day or equivalent dose). Control subjects had no history of asthma or wheeze, had no other chronic respiratory disease, had never smoked and their prebronchodilator FEV1 was ≥ 80% predicted. Expression values were plotted for each gene stratified by subject group. Data was analyzed using the Shapiro-Wilk test to test for normality before using the Kruskal-Wallis test (non-normal distribution) or Welch's ANOVA (normal distribution) to analyze differences between groups. Only genes GSDMB and STAT6 had normally distributed data. Both statistical tests were combined with a two-stage linear step-up procedure of Benjamini, Krieger, and Yekutieli to control the FDR at 5%. *P* < 0.05 was considered significant.

### Gene Functional Annotation Analysis

To identify gene or gene clusters in functional and disease related associations, the DAVID Bioinformatics Resource (31, 32) was used. The 37 candidate genes underwent enrichment analysis in the Genetic Association Database of complex diseases and disorders (GAD), Gene Ontology (GO) Term and KEGG and REACTOME pathway databases. The 37 genes were used as a set and clusters below 5% FDR were considered significant. The top 10 GAD terms were listed and fold enrichment (FE) scores were plotted against -log_10_(*P*-value) with the area of points representing the number of genes in the cluster for GO Terms and pathway analysis.

### Gene Interaction Analysis

To identify and predict candidate causal gene interactions, the GeneMANIA prediction server was used which normalizes and weights interaction networks from various sources of data to build an interaction map (33). The 37 candidate genes were inputted as a set to produce a network map depicting physical interactions, co-localization, co-expression and predicted genes and interactions. Scores for predicted genes and the number of gene interactions were used to identify the strongest predicted genes.

### Gene Drug Interaction Analysis

We used The Drug Gene Interaction Database (DGIdb) (34) to identify known drug interactions with the 37 genes highlighted by our signal to gene analysis and 5 predicted genes (*CD274, IL37, IRAK4, PDCD1LG2*, and *ZAP70*) which were found to have the strongest interactions through the gene interaction analysis. Drugs showing an interaction score, i.e., the numeric representation of publication count and source count, the ratio of average known gene partners for all drugs to the known partners for the given drug, and the ratio of average known drug partners for all genes to the known partners for the given gene, of >1.0 were selected. In addition we queried each gene at clinicaltrials.gov in order to identify drugs currently in Phase II, III, and IV trials for our identified genes as targets.

## Results

### PheWAS Analysis Indicates Signals Are Associated With Blood Cell Counts, Asthma, and Other Inflammatory Disorders

**Figure 2:**
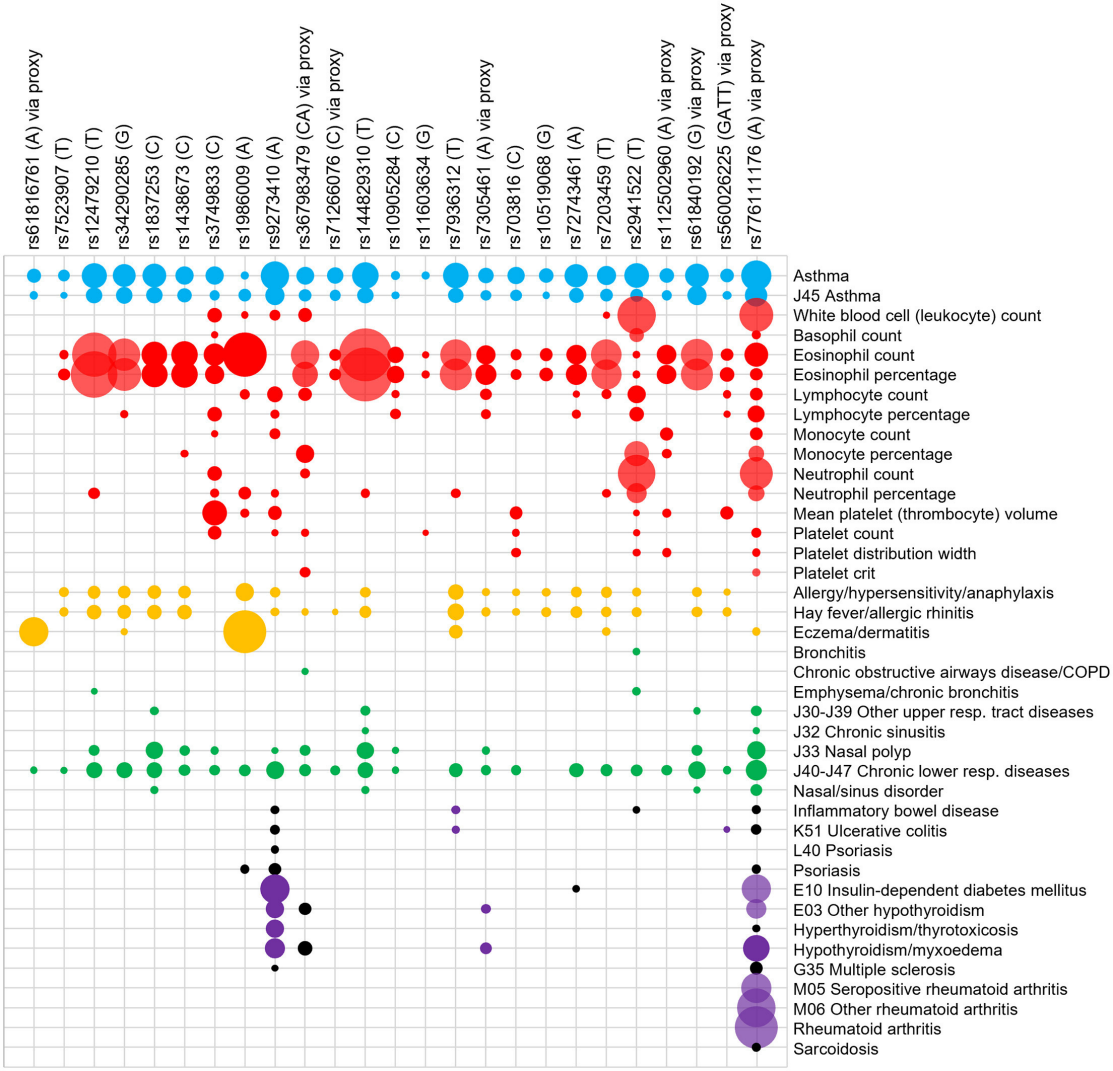
PheWAS of signals for moderate-severe asthma. Signals (risk allele in brackets) or their proxies were searched for in the GeneATLAS PheWAS database of the UK Biobank cohort. A Bonferroni adjusted *p*-value of 6.42 × 10^−5^ was used. The interaction between signals (top) and PheWAS traits (right) are represented on a grid and the area of the circle represents the -log_10_ (*p*-value) of the association. A larger area indicates a lower *p*-value. Only selected PheWAS traits have been displayed and organized into asthma (blue), blood/immune cell (red), allergy (yellow), other respiratory (green), inflammatory (purple), and auto-immune (gray) groups. The trait “basophil percentage” did not meet the Bonferroni corrected *P*-value for any of the signals and therefore was not included in the Figure. A full list of PheWAS terms and Beta and *P*-values are available in Supplementary Table 1. For disease traits (i.e., all except blood cell traits) a black dot represents an odds ratio of <1 with respect to the moderate to severe asthma risk allele. Proxies were used for the following signals which were not present in the database: rs61816761 (rs61816766, *R*^2^ = 0.50), rs367983479 (rs1504215, *R*^2^ = 0.85), rs71266076 (rs7824993, *R*^2^ = 0.81), rs7305461 (rs1131017, *R*^2^ = 0.75), rs112502960 (rs62076439, *R*^2^ = 1.0), rs61840192 (rs1031163, *R*^2^ = 1.0), rs560026225 (rs72687036, *R*^2^ = 0.66), and rs776111176 (rs3997872, *R*^2^ = 0.82).

The GeneATLAS database identified traits associated with the risk allele of the 25 signals or proxies from the Moderate to Severe Asthma GWAS (Tables 1, 2). As expected, all signals showed association with asthma with odds ratios (OR) >1 (Figure 2). All signals, apart from rs61816761 (*via* proxy rs61816766), showed association with a blood/immune cell traits particularly eosinophil levels but also lymphocyte and neutrophil counts.

**Table 1:** The 25 signals from the moderate to severe asthma GWAS.

**SNP**	**Closest gene reported in paper**	**Risk allele**	**Non-risk allele**	**Risk allele frequency in European population**
rs61816761	FLG	A	G	2.37%
rs7523907	CD247	T	C	54.08%
rs12479210	IL1RL1	T	C	38.73%
rs34290285	D2HGDH	G	A	74.26%
rs560026225^*^	KIAA1109	GATT	G	23.60%
rs1837253	TSLP	C	T	74.16%
rs1438673	WDR36	C	T	50.78%
rs3749833	C5orf56	C	T	26.08%
rs1986009	RAD50	A	C	18.71%
rs9273410	HLA-DQB1	A	C	55.30%
rs776111176^*^	HLA-DQA1	A	AAT	14.85%
rs367983479^*^	BACH2	CA	C	61.50%
rs71266076^*^	MIR5708	C	CT	36.93%
rs144829310	IL33	T	G	16.40%
rs10905284	GATA3	C	A	42.94%
rs11603634	MUC5AC	G	A	50.36%
rs7936312	C11orf30	T	G	47.42%
rs7305461	RPS26	A	C	44.61%
rs703816	STAT6	C	T	43.41%
rs10519068	RORA	G	A	87.25%
rs72743461	SMAD3	A	C	23.60%
rs7203459	CLEC16A	T	C	75.44%
rs2941522	IKZF3	T	C	48.29%
rs112502960	ZNF652	A	G	35.92%
rs61840192	LOC101928272	G	A	57.30%

*The 25 signals with the original reported gene (closest), asthma and non-risk allele and asthma risk allele frequency are listed. All analyses conducted in this study are based on the risk allele*.

* *Insertion and deletions (Indels)*.

**Table 2:** SNPs utilized in pipeline analyses.

**Variant**	**LD block /base pairs**	**Number of SNPs in LD (R^**2**^ >0.1)**	**Proxy used in other analyses (MAF)**	***R* ^**2**^**	**UBIOPRED Bronchial Biopsy (MAF)**	***R* ^**2**^**	**UBIOPRED Bronchial Brush (MAF)**	***R* ^**2**^**	**UBIOPRED Nasal Brush (MAF)**	***R* ^**2**^**
rs61816761	988,882	20	rs61816766 (C, 2%)	0.50	rs61816764 (T, 5%)	0.21	-	-	-	-
rs7523907	41,739	56	rs3108155 (G, 39%)	0.83	rs2056625 (A, 36%)	0.73	-	-	-	-
rs12479210	458,394	578	-	-	rs2270298 (G, 25%)	0.47	rs2241116 (A, 20%)	0.46	-	-
rs34290285	69,955	157	-	-	rs34077392 (C, 37%)	0.43	-	-	-	-
rs560026225	622,215^*^	150^*^	rs72687036 (G, 24%)	0.66	-	-	-	-	-	-
rs1837253	71,462	29	-	-	-	-	-	-	-	-
rs1438673	227,233	353	-	-	rs2289277 (G, 46%)	0.73	-	-	rs7524421 (G, 24%)	1.00
rs3749833	526,932	325	-	-	-	-	-	-	rs11748326 (T, 23%)	0.76
rs1986009	365,939	239	rs12652920 (C, 19%)	1.00	rs12652920 (C, 19%)	1.0	rs4705952 (G, 27%)	0.21	-	-
rs9273410	554,975	5,739	-	-	-	-	-	-	-	-
rs776111176	574,227^*^	1,447^*^	rs3997872 (A, 16%)	0.82	-	-	-	-	-	-
rs367983479	244,383	191	rs1504215 (A, 36%)	0.85	rs4142967 (T, 46%)	0.56	-	-	-	-
rs71266076^∧^	175,000	206	rs7824993 (A, 35%)	0.81	-	-	-	-	-	-
rs144829310	496,605	234	-	-	rs1929996 (C, 31%)	0.43	-	-	-	-
rs10905284	67,858	96	-	-	-	-	-	-	-	-
rs11603634	146,648	187	-	-	rs11602802 (C, 33%)	0.40	-	-	-	-
rs7936312	316,410	277	rs7936323 (A, 47%)	0.96	-	-	-	-	-	-
rs7305461	315,101	120	rs1131017 (C, 41%)	0.75	-	-	-	-	-	-
rs703816	284,371	165	-	-	rs167769 (T, 33%)	0.82	-	-	-	-
rs10519068	108,343	123	-	-	rs2279292 (C, 15%)	0.91	rs2279296 (G, 45%)	0.13	rs2279296 (G, 45%)	0.13
rs72743461	30,796	45	-	-	-	-	rs10152544 (C, 48%)	0.30	rs10152544 (C, 48%)	0.30
rs7203459	429,719	435	-	-	rs12919828 (G, 25%)	0.98	rs6498135 (A, 40%)	0.30	-	-
rs2941522	805,248	896	-	-	rs907091 (T, 49%)	0.96	-	-	-	-
rs112502960	270,023	370	rs62076439 (T, 36%)	1.00	-	-	-	-	-	-
rs61840192	201,710	249	rs1031163 (T, 42%)	1.00	-	-	-	-	-	-

*Each sentinel SNP reported in the original moderate to severe asthma GWAS corresponds to a haplotype consisting of related SNPs (R^2^ > 0.2) as identified in column 2. These SNPs provided us with potential proxies in cases where the original sentinel SNP was not covered by the in-silico platform and also gave an idea of the size of the LD pattern of the signal. In each case, the SNP with the highest R^2^ value available was selected as proxy. Multiple proxies were used in instances where an originally selected proxy was unavailable in one or more of the U-BIOPRED datasets. Where a proxy has not been stated (-) then the sentinel SNP was used*.

* *Indicates the sentinel SNP was not found in 1,000 Genomes Project accessed through LDLink (Phase 3) or HaploReg (Phase 1), ergo values are reflective of the proxy SNP used*.

∧ *This sentinel SNP (rs71266076) was not found in the 1,000 Genomes Project Phase 3 data accessed through LDLink therefore Phase 1 data accessed through HaploReg was used instead*.

*MAF, minor allele frequency in the European population from 1,000 Genomes Project Phase 3*.

Signal rs11603634 was relatively specific in showing only asthma, eosinophil percentage and platelet count associations. All signals apart from rs61816761 and rs9273410 showed association with eosinophil number and/or percentage highlighting the role of this cell type in asthma. All signals apart from rs61816761, rs3749833, rs10905284, rs11603634, rs112502960, and rs776111176 showed an association with allergy. Of these, signals rs3749833, rs10905284, rs11603634, rs112502960, and rs776111176 were associated with eosinophils but not allergy. Conversely rs9273410 was the only signal which showed an association with allergy but not eosinophils. These nuances in associations highlight the different asthma phenotypes.

Signals, rs1986009, rs9273410, rs7936312, rs776111176, and rs2941522 showed broad associations across many trait and trait groups. Signals rs9273410, rs776111176, and rs72743461 were the only ones associated with insulin-dependent diabetes. Signal rs776111176 was the only one associated with rheumatoid arthritis.

Some signals showed protective associations (OR < 1, black dots) for other disease traits such as rs2941522 (inflammatory bowel disease), rs72743461 (diabetes), rs1986009 (psoriasis) rs776111176 (inflammatory bowel diseases, ulcerative colitis, psoriasis, hyperthyroidism, multiple sclerosis, sarcoidosis), and rs9273410 (inflammatory bowel diseases, ulcerative colitis, psoriasis and multiple sclerosis). A full list of traits and ORs and *P*-values are available in Supplementary Table 1.

### Signal to Gene Analysis Identifies 37 Candidate Causal Genes Associated With the 25 Moderate-Severe Association Signals

Using our scoring system for identifying candidate causal genes (Table 3) we identified 33 moderate to strong signal:gene relationships (s ≥ 3) for 21/25 signals (Table 4). The four remaining signals presented with weaker evidence of a relationship (s ≤ 2).

**Table 3:** Scoring system for signal to gene analyses.

**Evidence**	**Weighting**
GTEX respiratory relevant cell/tissue eQTL (*P* < 0.05)	1
GTEX Blood eQTL (*P* < 0.05)	1
GTEX respiratory relevant cell/tissue sQTL (*P* < 0.05)	1
GTEX blood sQTL (*P* < 0.05)	1
OTG eQTL Resp (*P* < 0.05)	1
OTG eQTL blood (*P* < 0.05)	1
OTG V2G score (presence of)	1
OTG co-localization study (H4 > 0.8)	1
HaploReg eQTL (*P* < 0.05)	1
Functional variant (presence of in an LD *r*^2^ > 0.1)	1
UBIOPRED brush eQTL (*P* < 0.05)	2
UBIOPRED biopsy eQTL (*P* < 0.05)	2
UBIOPRED nasal eQTL (P < 0.05)	2
Literature association (presence of)	1
**Total possible score**	**16**

*Each potential candidate causal gene was scored based on supporting evidence. eQTL and sQTL associations were reported on presenting a p-value of <0.05, co-localization studies on presenting an H4 > 0.8 and functional variants if present withing a linkage disequilibrium block with an r^2^ > 0.1. Double weighting was given to eQTL associations observed in U-BIOPRED, due to the increased relevance of lung tissue and airway epithelial tissues to our study*.

**Table 4:** Selection of genes of interest relative to signal of association.

**SNP**	**Gene in paper**	**Identified genes**	**Score**	**UB score**	**Total score**
rs61816761	FLG	FLG	2	0	2
		TUFT1	0	1	2
		SELENBP1	0	1	2
		C1orf68	0	1	2
rs7523907	CD247	CD247	7	0	7
		BRP44	0	1	2
		CREG1	0	1	2
rs12479210	IL1RL1	IL1RL1	11	1	13
		IL1R1	0	1	2
		IL18R1	6	0	6
rs34290285	D2HGDH	D2HGDH	11	1	13
		PDCD1	3	0	3
		GAL3ST2	1	0	1
		KIF1A	1	1	3
rs560026225	KIAA1109	KIAA1109	4	0	4
rs1837253	TSLP	TSLP	2	0	2
		WDR36	1	0	1
rs1438673	WDR36	WDR36	3	0	3
		CAMK4	1	0	1
		TMEM232	0	1	2
		TSLP	1	1	3
		STMN1	0	1	2
rs3749833	C5orf56	SLC22A5	8	0	8
		SLC22A4	2	0	2
		P4HA2	2	0	2
		C5orf56	1	0	1
		IRF1	3	0	3
		LOC553103	0	1	2
		SEPT8	0	1	2
		ANKRD43	0	1	2
rs1986009	RAD50	SLC22A5	3	0	3
		SLC22A4	3	0	3
		ACSL6	0	1	2
		IL13	0	1	2
		IL4	0	1	2
rs9273410	HLA-DQB1	HLA-DQB1	2	3	8
		HLA-DQB2	2	3	8
		HLA-DQA1	2	0	2
		HLA-DQA2	2	0	2
		ATF6B	1	0	1
rs776111176	HLA-DQA1	HLA-DQA1	6	2	8
		HLA-DQA2	6	0	6
		HLA-DQB1	6	6	12
		HLA-DQB2	6	0	6
		HLA-DRB3	0	2	2
		HLA-DOB	4	0	4
		AGER	0	2	2
		LY6G6E	0	2	2
		PFDN6	0	2	2
		NEU1	0	2	2
		DOM3Z	0	2	2
		LY6G6D	0	2	2
		COL11A2	0	2	2
rs367983479	BACH2	BACH2	3	0	3
		ANKRD6	0	1	2
		MAP3K7	0	1	2
		GABRR2	0	1	2
rs71266076	MIR5708	ZBTB10	1	0	1
rs144829310	IL33	IL33	2	2	6
		ERMP1	1	0	1
		TPD52L3	0	1	2
rs10905284	GATA3	GATA3	2	0	2
rs11603634	MUC5AC	MUC5AC	2	2	6
		TNNT3	0	1	2
rs7936312	C11orf30	LRRC32	4	0	4
		BRCA2	1	0	1
rs7305461	RPS26	RPS26	4	0	4
		SUOX	4	0	4
		RAB5B	2	0	2
		ERBB3	2	0	2
		ESYT1	1	0	1
		GDF11	1	0	1
		RNF41	1	0	1
rs703816	STAT6	STAT6	5	0	5
		NEMP1	1	0	1
		RBMS2	1	0	1
		SPRYD4	1	0	1
		EEF1AKMT3	1	0	1
		ZBTB39	0	1	2
		CDK4	0	1	2
		ESYT1	0	1	2
rs10519068	RORA	RORA	4	0	4
		ICE2	0	1	2
		ANXA2	0	1	2
		FOXB1	0	1	2
s72743461	SMAD3	SMAD3	4	0	4
		AAGAB	3	0	3
		C15orf61	0	1	2
		BPGM	0	1	2
		MAP2K1	0	1	2
rs7203459	CLEC16A	CLEC16A	2	1	4
		TEKT5	0	1	2
		PRM1	0	1	2
		DEXI	3	0	3
rs2941522	IKZF3	ORMDL3	4	0	4
		GSDMB	5	0	5
		GSDMA	2	0	2
		PGAP3	3	0	3
		MSL1	1	1	3
		IKZF3	2	0	2
		ARL5C	0	1	2
rs112502960	ZNF652	ZNF652	3	0	3
		GNGT2	3	0	3
		PHOSPHO1	2	0	2
		TMEM92	0	1	2
		HOXB4	0	1	2
		NCRNA00253	0	1	2
rs61840192	LOC101928272	GATA3	4	0	4
		GATA3-AS1	2	0	2

*Signals were analyzed using the translational pipeline including publicly available datasets such as Open Target Genetics, GTex, HaploReg and LDLink and our UBIOPRED (UB) datasets of Bronchial Biopsies, Bronchial Brushes, and Nasal Brushes. Each gene was scored based on supporting evidence as laid out in Table 3, with double weighting being given to eQTL associations observed in UB, based on the datasets localized data. The genes highlighted in green were taken forward in downstream analyses*.

Relationships with the greatest score were observed for the signals tagged by rs12479210 (*IL1RL1*) and rs34290285 (*D2HGDH*) (score = 13) and multiple genes were identified for signals rs12479210, rs34290285, rs1438673, rs3749833, rs1986009, rs7305461, rs72743461, rs7203459, rs776111176, rs2941522, and rs112502960. We identified novel candidate causal signal:gene relationships for SNPs rs3749833, rs1986009, rs71266076, rs7936312, and rs2941522.

**Figure 3:**
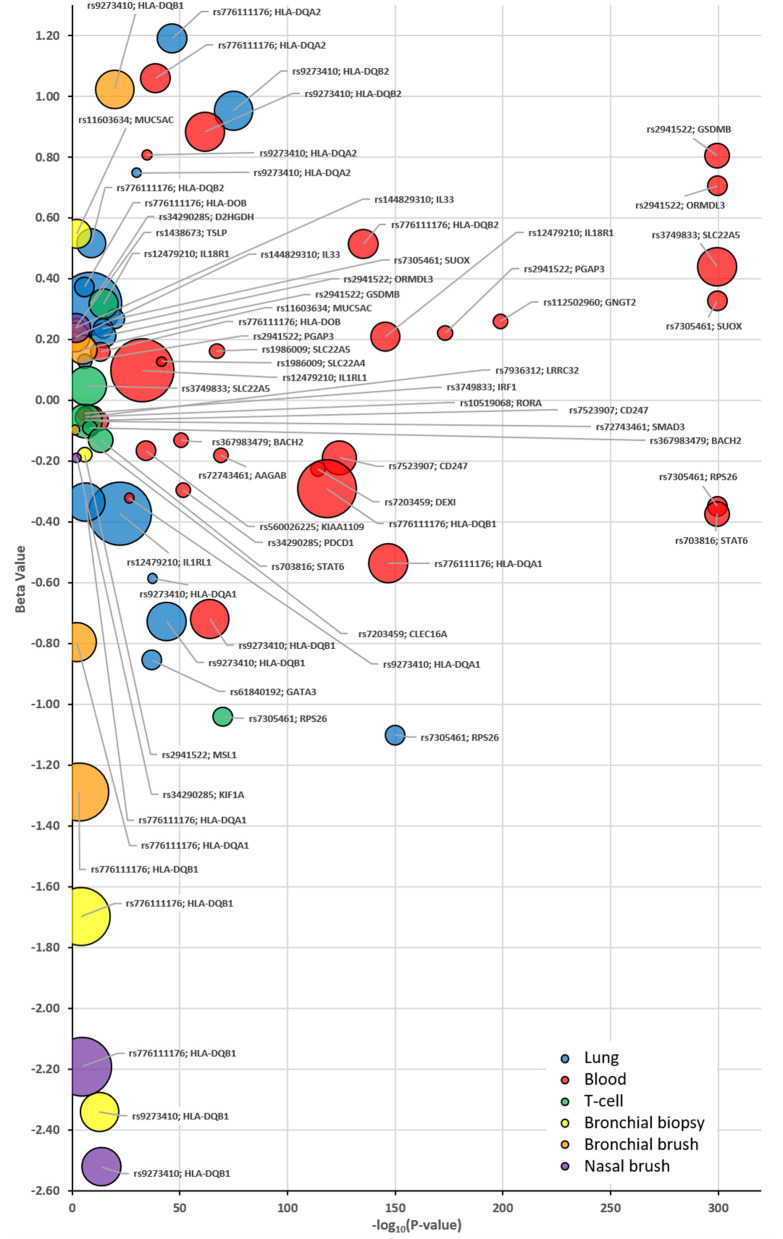
Bubble plots identifying the eQTL patterns for each selected SNP:Gene pairing as identified in Table 3 and tissue specific eQTLs. Plot identifies each eQTL *P*-value (x-axis) and *B*-value (y-axis) stratified based on the asthma risk allele for each identified compartment as denoted by the corresponding color; Blue = Lung, Red = Whole Blood, Green = T-cells, Yellow = U-BIOPRED Bronchial Biopsy, Orange = U-BIOPRED Bronchial Brush and Purple—U-BIOPRED Nasal Brush. The size of the bubble indicates the generated score in Table 3. EQTLs listed are those that achieved a *P*-value of *P* < 0.05 in one of the interrogated datasets.

The strongest eQTLs, as defined by the most significant combined *P* and B-values for the risk allele, were observed in whole blood for rs2941522 (*GSDMB* & *ORMDL3*) and rs7305461 (*RPS26* & *STAT6*) (Figure 3). Weaker associations (*P*-value) presenting with the greatest observed effect (B-value) were observed for rs776111176 in whole blood, lung (*HLA-DQA2*) and for signal rs9273410 in bronchial brushes (*HLA-DQB1*) and in whole blood and lung (*HLA-DQB2*) (Figure 3).

### Gene Function and Biological Processes Analysis Identifies Distinct Gene Groups

The largest gene function clusters were observed for genes involved in DNA/RNA binding (*BACH2, GATA3, IRF1, RORA, RPS26, SMAD3, STAT6*, and *WDR36*) or signal transduction (*CD247, GNGT2, HLA-DQA1/A2/B1/B2, IL18R1, IL1RL1, IL33, LRRC32, PDCD1*, and *TSLP*). Both *FLG* and *MUC5AC* are structural components involved in barrier formation and GSDMB is involved in cell death. Some genes such as *DEXI, KIF1A, KIAA1109, ZBTB10*, and *ZNF652* had very limited or no information and as yet their function or involvement in biological processes is unknown. The gene function of implicated genes is described in Table 5.

**Table 5:** Gene function of candidate causal genes.

**Gene**	**Full name**	**Molecular function/biological process**
**Barrier formation/defense**		
*FLG*	Filaggrin	(1) protein binding (2) structural constituent of skin epidermis/establishment of skin barrier (3) peptide cross-linking
*MUC5AC*	Mucin 5AC, oligomeric mucus/gel-forming	(1) phosphatidylinositol-mediated signaling (2) gel-forming secretory mucin
**Cell death**		
*GSDMB*	Gasdermin B	(1) wide pore channel activity (2) granzyme-mediated programmed cell death signaling pathway (3) pyroptosis
**DNA/RNA binding**		
*BACH2*	BTB domain and CNC homolog 2	(1) sequence-specific double-stranded DNA binding
*GATA3*	GATA binding protein 3	(1) transcription regulatory region sequence-specific DNA binding (2) core promoter proximal region sequence-specific DNA binding (3) E-box binding (4) negative regulation of mammary gland epithelial cell proliferation (5) positive regulation of transcription, DNA-templated (6) positive regulation of transcription from RNA polymerase II promoter (7) mesenchymal to epithelial transition (8) lymphocyte migration (9) positive regulation of interleukin-5 and−13 secretion
*IRF1*	Interferon regulatory factor 1	(1) protein binding (2) RNA polymerase II core promoter proximal region sequence-specific DNA binding (3) apoptotic process (4) cell cycle arrest (5) cellular response to interferon-beta (6) defense response to virus
*RORA*	RAR related orphan receptor A	(1) DNA-binding transcription factor activity (2) ligand-activated transcription factor activity (3) oxysterol binding (4) RNA polymerase II cis-regulatory region sequence-specific DNA (5) cellular response to sterol (6) intracellular receptor signaling pathway (7) positive regulation of transcription, DNA-templated (8) positive regulation of transcription by RNA polymerase II
*RPS26*	Ribosomal protein S26	(1) negative regulation of RNA splicing (2) cadherin binding involved in cell-cell adhesion (3) mRNA binding (4) protein binding (5) structural constituent of ribosome (6) cytoplasmic translation (7) negative regulation of RNA splicing
*SMAD3*	SMAD family member 3	(1) RNA polymerase II core promoter proximal region sequence-specific DNA binding (2) transforming growth factor beta receptor binding and signaling pathway (3) zinc ion binding (4) phosphatase binding (5) protein kinase binding (6) ubiquitin protein ligase binding (7) protein homodimerization activity (8) negative regulation of transcription from RNA polymerase II promoter (9) positive regulation of epithelial to mesenchymal transition (10) evasion or tolerance of host defenses by virus (11) negative regulation of cell growth (12) positive regulation of transcription factor import into nucleus (13) positive regulation of nitric oxide biosynthetic process (14) negative regulation of fat cell differentiation (15) negative regulation of cytosolic calcium ion concentration (16) positive regulation of extracellular matrix assembly
*STAT6*	Signal transducer and activator of transcription 6	(1) protein binding (2) protein phosphatase binding (3) DNA-binding transcription activator activity, RNA polymerase II-specific
*WDR36*	WD repeat domain 36	(1) poly(A) RNA binding
**Membrane binding/transport**		
*AAGAB*	Alpha and gamma adaptin binding protein	(1) protein binding (2) protein transport (3) may be involved in endocytic recycling of growth factor receptors such as EGFR
*CLEC16A*	C-type lectin domain containing 16A	(1) possible involvement in autophagy and endosomal transport
*SLC22A4*	Solute carrier family 22 member 4	(1) carnitine transport (2) quaternary ammonium group transport (3) carnitine transmembrane transporter activity (4) cation:cation antiporter activity (5) PDZ domain binding (6) amino acid import across plasma membrane
*SLC22A5*	Solute carrier family 22 member 5	(1) carnitine transport (2) quaternary ammonium group transport (3) carnitine transmembrane transporter activity (4) PDZ domain binding (5) sodium-dependent organic cation transport
*ORMDL3*	ORMDL sphingolipid biosynthesis regulator 3	(1) Protein binding (2) positive regulation of autophagy (3) positive regulation of protein localization to nucleus (4) may indirectly regulate ER-mediated calcium signaling
**Metabolic processes**		
*D2HGDH*	D-2-hydroxyglutarate dehydrogenase	(1) (R)-2-hydroxyglutarate dehydrogenase activity
*PGAP3*	Post-GPI attachment to proteins phospholipase 3	(1) Possible involvement in GPI anchor metabolic process and hydrolase activity, acting on ester bonds
*MSL1*	MSL complex subunit 1	(1) Histone H4-K16 acetylation
*SUOX*	Sulfite oxidase	(1) Possible involvement in sulfur compound metabolic processing
**Signal transduction**		
*CD247*	CD247 molecule	(1) Protein binding (2) identical protein binding (3) protein tyrosine kinase binding
*GNGT2*	G protein subunit gamma transducin 2	(1) GTPase activity
*HLA-DQA1/A2/B1/B2*	Major histocompatibility complex, class II, DQ beta 1	(1) Humoral immune response mediated by circulating immunoglobulin (2) immunoglobulin production involved in immunoglobulin mediated immune response (3) antigen processing and presentation of exogenous peptide antigen *via* MHC class II (4) T cell receptor signaling pathway
*IL18R1*	Interleukin 18 receptor 1	(1) Protein binding (2) interleukin-18-mediated signaling pathway (3) positive regulation of interferon-gamma production (4) positive regulation of NF-kappaB transcription factor activity (5) positive regulation of NIK/NF-kappaB signaling (6) positive regulation of T-helper 1 cell cytokine production
*IL1RL1*	Interleukin 1 receptor like 1	(1) protein binding (2) IL33 receptor
*IL33*	Interleukin 33	(1) positive regulation of chemokine secretion (2) positive regulation of macrophage activation (3) cytokine activity (4) protein binding
*LRRC32*	Leucine rich repeat containing 32	(1) TGF-beta binding and signaling pathway
*PDCD1*	Programmed cell death 1	(1) protein binding (2) cell surface receptor signaling pathway (3) negative regulation of CD4-positive, alpha-beta T cell proliferation (4) positive regulation of activated CD8-positive, alpha-beta T cell apoptotic process (5) response to cytokine (6) signal transduction (7) T cell co-stimulation
*TSLP*	Thymic stromal lymphopoietin	(1) cytokine activity (2) positive regulation of tyrosine phosphorylation of STAT protein (3) promotes T helper type 2 cell responses (4) positive regulation of chemokine production (5) positive regulation of cytokine-mediated signaling pathway
**Unknown**		
*DEXI*	DEXI homolog	Unknown
*KIAA1109*	KIAA1109	(1) Protein binding
*KIF1A*	Kinesin family member 1A	(1) Identical protein binding
*ZNF652*	Zinc finger protein 652	(1) Protein binding
*ZBTB10*	Zinc finger and BTB domain containing 10	(1) Protein binding

*Gene function and biological process for the candidate genes as determined using the Gene 2 Function and UniProt databases, listing associated disease states and gene function annotations*.

### Single Cell RNA Expression in Lung Tissue Indicates Cell Type Specificity in Healthy Individuals

**Figure 4:**
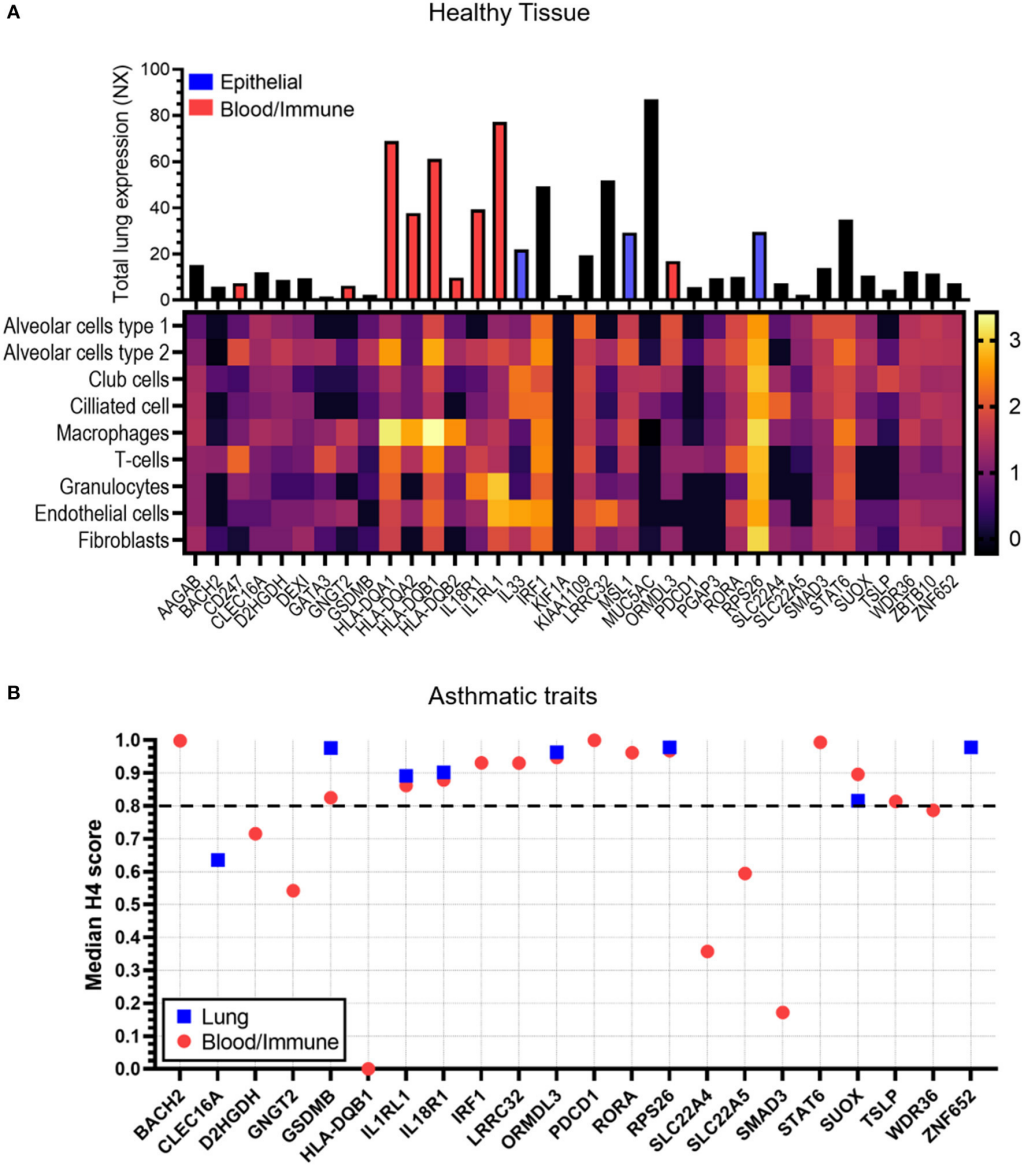
Tissue expression and colocalization analysis of candidate genes. **(A)** Data was taken from the Human Protein Atlas for both total lung RNA expression scores (bar chart, NX) and single cell RNA expression [heatmap, log_10_ (median transcripts per million protein coding genes)] in healthy tissue. Bar directly corresponds to gene in heatmap. The Human Protein Atlas was also analyzed for genes which showed enriched/enhanced gene expression in epithelial cells (blue bar) and blood/immune cells (red bar) compared to other cell types. A full list of enriched/enhanced tissue and cell gene expression data per gene is available in Supplementary Table 2. No data was available for FLG and therefore has not been shown. **(B)** Co-localization analysis (H4 scores) data was taken from the Open Target Genetics (OTG) platform for lung (blue squares) or blood/immune cells (red circles) in studies with the asthma trait (exclusive). Scores represent evidence of association between candidate gene, specific tissue and asthma trait. A higher score indicates greater association. Dotted line shows cut off value of 0.8. Candidate genes *AAGAB, CD247, DEXI, FLG, GATA3, HLA-DQA1/A2, B2, IL33, KIF1A, KIAA1109, MSL1, MUC5AC, PGAP3, and ZBTB10* had no association data for lung/blood/immune cells/tissue in OTG and therefore have not been shown.

The HPA resource was used to look at overall and single cell type gene expression profile in lung tissue from healthy donors for the 37 candidate causal genes. Genes which showed enriched/enhanced expression (4-fold higher expression than in other cells/tissues) in epithelial or blood/immune cells were also identified. All genes except *FLG* showed expression in lung tissue and cells (Figure 4). *KIF1A* showed lower levels of lung expression and was undetectable in the cell types analyzed potentially due to the sensitivity of this technique. *MUC5AC* showed overall highest expression in lung tissue and along with *IL33* and *SLC22A4* was enriched in club and ciliated cells. *IL33* also showed high expression in endothelial cells. Both *IRF1* and *RPS26* showed high lung cell type expression but no cell type specificity. *CD247, GNGT2, HLA-DQB2*, and *PDCD1* were expressed at low levels in the datasets and were specific for T-cells and macrophages. *HLA-DQA1/A2, HLA-DQB1* were highly expressed overall and specifically in macrophages, whereas *IL18R1* and *IL1RL1* showed greater expression in granulocytes. A full list of enriched tissues and cell types is available in Supplementary Table 2.

### Co-localization Analysis Reveals Genes With High Asthma Trait Associations in Lung Tissues and Blood/Immune Cells

The OTG platform was used to investigate whether gene expression in lung tissue and/or blood immune/cells was associated with the asthma trait (Figure 4B). *BACH2, IRF1, LRRC32, PDCD1, RORA, STAT6, and TSLP* showed a strong link (H4 score>0.8) to blood/immune cells only, whereas *ZNF652* was exclusively linked to lung tissue expression. Genes *GSDMB, IL1RL1, IL18R1, ORMDL3, RPS26*, and *SUOX* on the other hand showed strong association to both lung tissue and blood/immune cells.

### Genes Show Differential Gene Expression in Bronchial Epithelial Cells Isolated From Subjects With Severe Asthma

**Figure 5:**
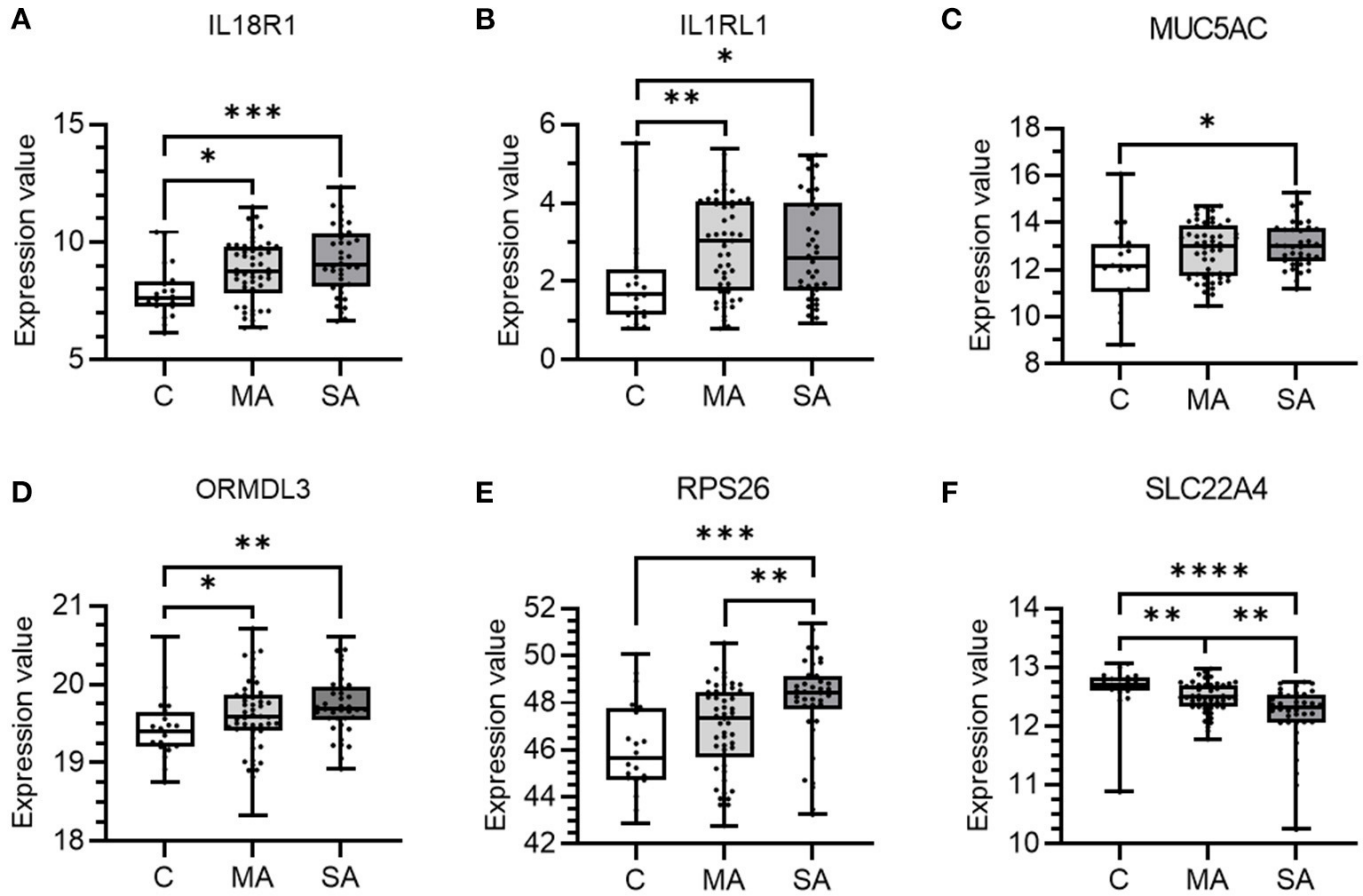
mRNA expression of candidate causal genes in bronchial epithelial cells taken from patients with asthma and controls. Boxes shows the median and IQR and the whiskers show the minimum and maximum data. Bronchial epithelial brush samples were from controls (C, *n* = 20) and patients with mild-moderate (MA, *n* = 50) and severe (SA, *n* = 38) asthma. Data is shown for **(A)** IL18R1, **(B)** IL1RL1, **(C)** MUC5AC, **(D)** ORMDL3, **(E)** RPS26, and **(F)** SLC22A4. Expression values were taken from the dataset and a Kruskal-Wallis test with a two-stage linear step-up procedure of Benjamini, Krieger and Yekutieli used to control the FDR at 5% was performed. *P < 0.05, **P < 0.01, ***P < 0.001, and ****P < 0.0001.

From the above analyses, 10 genes (*GSDMB, IL18R1, IL1RL1, IL33, MUC5AC, ORMDL3, RPS26, SLC22A4, SUOX, and ZNF652)* showed enriched epithelial expression and/or co-localization of the asthma trait in lung tissue. These specific genes were further investigated in the bronchial epithelium of mild-moderate or severe asthma subjects compared to control subjects (Figure 5). Six of the 10 studied genes showed differential expression in asthma vs. control subject cells. Expression levels of *IL18RL1* (*P* = 0.019, MA and 0.0008, SA), *IL1RL1* (*P* = 0.0034, MA and 0.010, SA), and *ORMDL3* (*P* = 0.040, MA and 0.0029, SA) were higher in both mild-moderate and severe asthma subjects compared to controls (Figures 5A,B,D) whereas expression levels of *MUC5AC* (*P* = 0.022) and *RPS26* (*P* = 0.0004) were higher in severe asthma subjects only compared to controls (Figures 5C,E). Expression levels of *SLC22A4* (*P* = 0.0051, MA and <0.0001, SA) was lower in both mild-moderate and severe asthma subjects compared to controls (Figure 5F). Expression levels of *SLC22A4* (*P* = 0.0028) was decreased in severe asthma (Figure 5F) compared to mild-moderate asthma. Genes *GSDMB, IL33, SUOX*, and *ZNF652* did not show any significant differences in expression across groups (Supplementary Figure 1).

### Genes Show Differential Gene Expression in Blood Cells Isolated From Subjects With Severe Asthma

**Figure 6:**
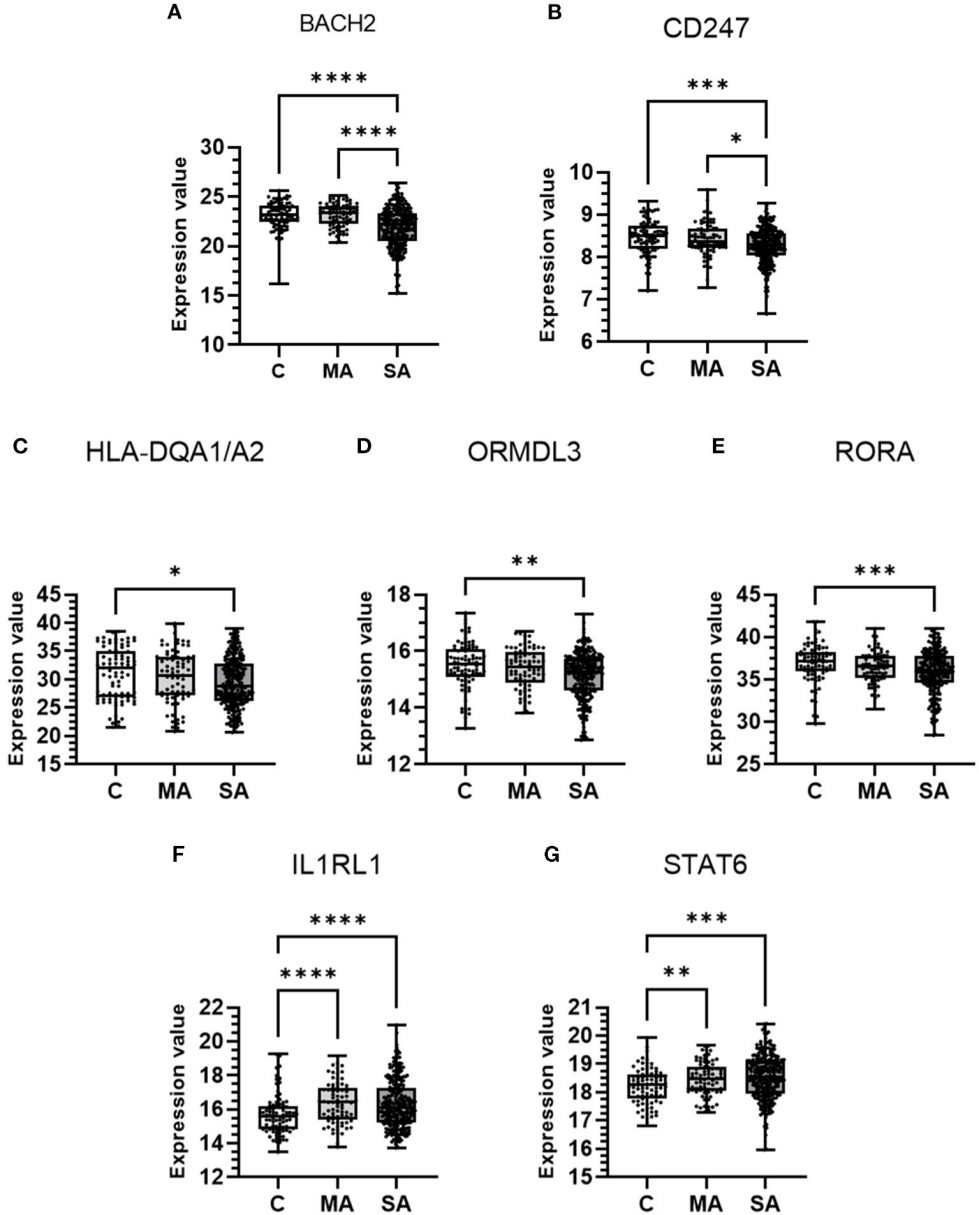
mRNA expression of candidate causal genes in blood cells taken from patients with asthma and controls. Boxes shows the median and IQR and the whiskers show the minimum and maximum data. Blood samples were from controls (C, *n* = 87) and patients with mild-moderate (MA, *n* = 77) and severe (SA, *n* = 246) asthma. Data is shown for **(A)** BACH2, **(B)** CD247, **(C)** HLA-DQA1/A2, **(D)** ORMDL3, **(E)** RORA, **(F)** IL1RL1, and **(G)** STAT6. Expression values were taken from the dataset and either a Kruskal-Wallis test or Welch's ANOVA (STAT6), both with a two-stage linear step-up procedure of Benjamini, Krieger and Yekutieli used to control the FDR at 5%, was performed. *P < 0.05, **P < 0.01, ***P < 0.001, and ****P < 0.0001.

From the above analyses, 18 genes (*BACH2, CD247, HLA-DQA1/A2, IL1RL1, ORMDL3, RORA, STAT6, GNGT2, GSDMB, HLA-DQB1/B2, IL18R1, IRF1, LRRC32, PDCD1, RPS26, SUOX*, and *TSLP*) showed enriched blood/immune expression and/or co-localization of the asthma trait in blood. These genes were further investigated in blood cells of mild-moderate or severe asthma subjects compared to control subject cells (Figure 6). Seven of the 18 genes studied showed differential expression across control and asthma subject groups. Expression levels of *BACH2* (*P* = <0.0001 for both) and *CD247* (*P* = 0.0002, vs. C and *P* = 0.0204 vs. MA for *CD247*) were significantly lower in severe asthma compared to controls and mild-moderate asthma (Figures 6A,B). Expression levels of *HLA-DQA1/A2* (*P* = 0.0077), *ORMDL3* (*P* = 0.0021), and *RORA* (*P* = 0.0005) were significantly lower in severe asthma subjects only compared to controls (Figures 6C–E). Expression levels of *IL1RL1* (*P* = 0.0002 vs. MA, *P* = <0.0001 vs. SA) and *STAT6* (*P* = 0.0079 vs. MA, *P* = <0.0001 vs. SA) were significantly higher in mild-moderate and severe asthma compared to controls (Figures 6F,G). Genes *GNGT2, GSDMB, HLA-DQB1/B2, IL18R1, IRF1, LRRC32, PDCD1, RPS26, SUOX*, and *TSLP* didn't show any significant differences in expression between groups (Supplementary Figure 2).

### Genetic Association Database of Complex Diseases and Disorders Identifies Genes Associated With Asthma

The DAVID functional annotation tool was used to perform enrichment analysis for the 37 candidate genes in the GAD. The top 10 gene clusters (5% FDR) are listed in Table 6. The full table is available in Supplementary Table 3. Asthma was significantly associated with the largest cluster of genes comprising of *FLG, IL33, GSDMB, SLC22A5, SMAD3, TSLP, GATA3, SUOX, MUC5AC, IL1RL1, IRF1, ORMDL3, STAT6, HLA-DQA2, IL18R1, HLA-DQA1*, and *HLA-DQB1* (*P* = 1.78 × 10^−15^). Genes were also associated with other, predominantly autoimmune, diseases including Type 1 diabetes, Celiac disease, Crohn's disease, and rheumatoid arthritis which complements the findings of the PheWAS. There is considerable overlap between genes associated with asthma and other diseases with *HLA-DQA1/B1* genes present in ~74% of significant terms (Supplementary Table 4).

**Table 6:** DAVID functional annotation tool analysis of the genetic association database of complex diseases and disorders (GAD).

**Term**	**Genes**	**Count (%)**	FE^*^	***p*-value**	**FDR**
Asthma	FLG, IL33, GSDMB, SLC22A5, SMAD3, TSLP, GATA3, SUOX, MUC5AC, IL1RL1, IRF1, ORMDL3, STAT6, HLA-DQA2, IL18R1, HLA-DQA1, HLA-DQB1	17 (45.95)	14.53	1.78 × 10^−15^	1.07 × 10^−12^
Diabetes mellitus, type 1	KIAA1109, GSDMB, CLEC16A, PDCD1, SUOX, HLA-DQA2, BACH2, HLA-DQA1, HLA-DQB1	9 (24.32)	32.76	1.58 × 10^−10^	4.76 × 10^−08^
Celiac disease	IL1RL1, KIAA1109, IRF1, CLEC16A, CD247, BACH2, IL18R1, HLA-DQA1, HLA-DQB1	9 (24.32)	22.97	2.72 × 10^−09^	5.44 × 10^−07^
Nasal polyposis	IL33, IL1RL1, WDR36, HLA-DQA1, HLA-DQB1	5 (13.51)	140.38	3.00 × 10^−08^	4.30 × 10^−06^
Obesity|asthma	IL33, IL1RL1, GSDMB, TSLP, ORMDL3, IL18R1	6 (16.22)	60.47	3.57 × 10^−08^	4.30 × 10^−06^
Ulcerative colitis	SLC22A4, GSDMB, SLC22A5, ORMDL3, STAT6, HLA-DQA1, HLA-DQB1	7 (18.92)	31.27	6.45 × 10^−08^	6.46 × 10^−06^
Arthritis, rheumatoid	SLC22A4, IL1RL1, KIAA1109, CD247, HLA-DQA2, HLA-DQA1, HLA-DQB1	7 (18.92)	19.79	9.79 × 10^−07^	8.41 × 10^−05^
Crohn's disease	GSDMB, SLC22A5, SMAD3, ORMDL3, BACH2, HLA-DQA1	6 (16.22)	23.12	4.63 × 10^−06^	3.48 × 10^−04^
Diabetes, type 1	SLC22A4, SLC22A5, IRF1, CLEC16A, PDCD1, HLA-DQA1, HLA-DQB1	7 (18.92)	14.48	6.02 × 10^−06^	3.62 × 10^−04^
Rheumatoid arthritis	SLC22A4, SLC22A5, CLEC16A, PDCD1, HLA-DQB2, HLA-DQA1, HLA-DQB1	7 (18.92)	14.48	6.02 × 10^−06^	3.62 × 10^−04^

*The top 10 genes clusters and disease associations are shown, the full table is available in Supplementary Table 3. All results are below 5% FDR*.

* *Fold enrichment*.

### Pathway and Gene Ontology Term Enrichment Analysis Highlights Genes Involved in Asthma and the Strong Presence of a HLA-DQA1/A2/B1/B2 Cluster

**Figure 7:**
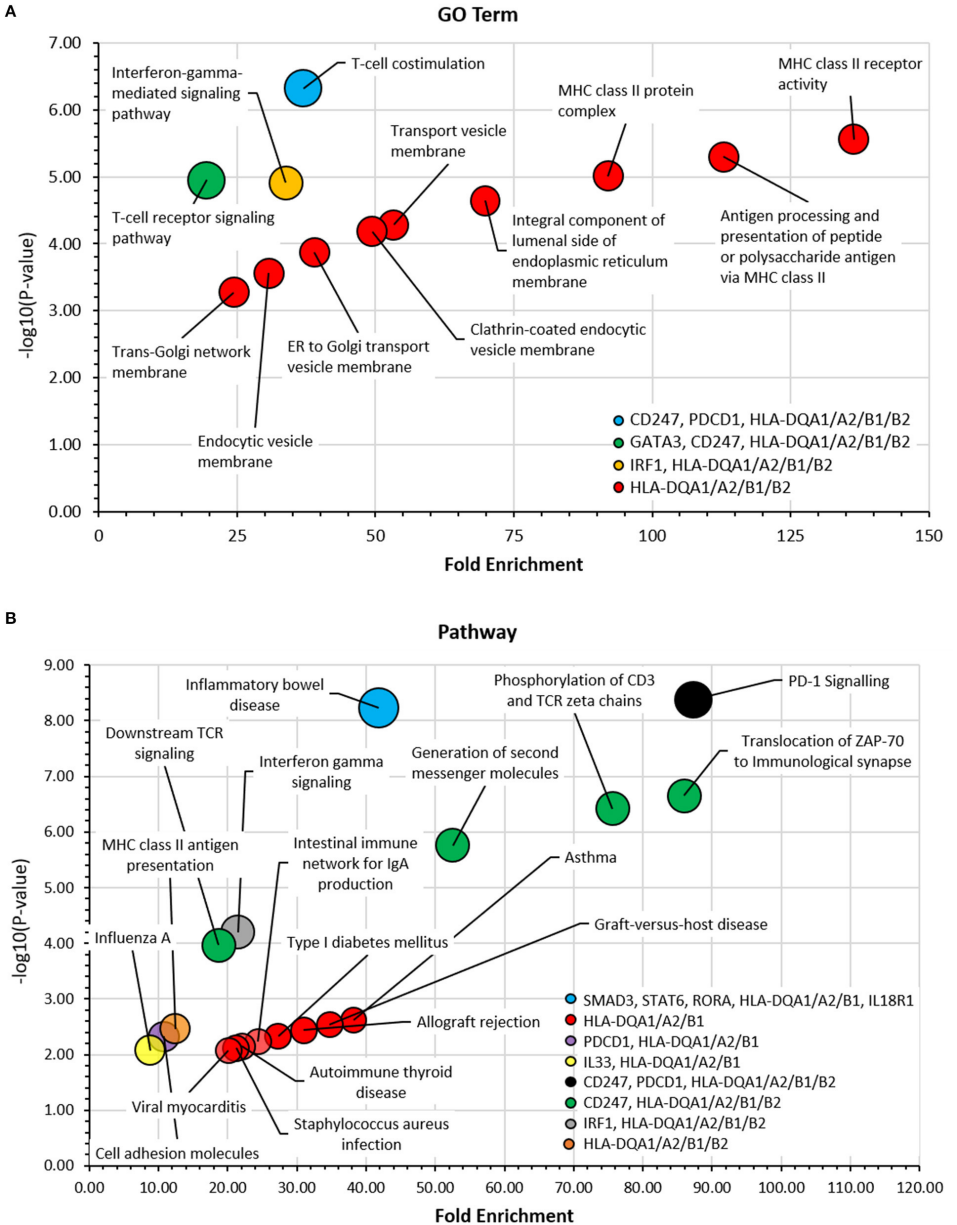
Gene ontology (GO) term and pathway analyses. The DAVID bioinformatic tools was used to assess candidate genes in **(A)** GO Term and **(B)** KEGG and REACTOME Pathway analyses. -log_10_ (*p*-value) was plotted against fold enrichment for gene groups and the area of the circle is proportional to number of genes in the group. 5% FDR. Full analyses can be viewed in Supplementary Tables 5, 6.

GO term enrichment analysis (Figure 7A) identified a predominant gene cluster of *HLA-DQA1/A2/B1/B2* genes. These genes were enriched mainly in processes involving MHC class II molecules and vesicle-membrane transport interactions. KEGG and REACTOME pathway analyses also identified this gene cluster as being involved in asthma, MHC class II antigen presentation, autoimmune processes, pathogenic infections, and diabetes (Figure 7B). PD-1 signaling was the most significant pathway identified which was enriched for *CD247, PDCD1, HLA-DQA1/A2*, and *HLA-DQB1/2*. Other significant pathways, including phosphorylation, translocation and generation of second messenger molecules, involved the *CD247, HLA-DQA1/A2*/*B1/B2* genes. Full result tables are available in Supplementary Tables 5, 6.

### Gene Interactions Analysis Highlights Key Co-localization and Co-expression Relationships and Predicts Additional Related Genes

**Figure 8:**
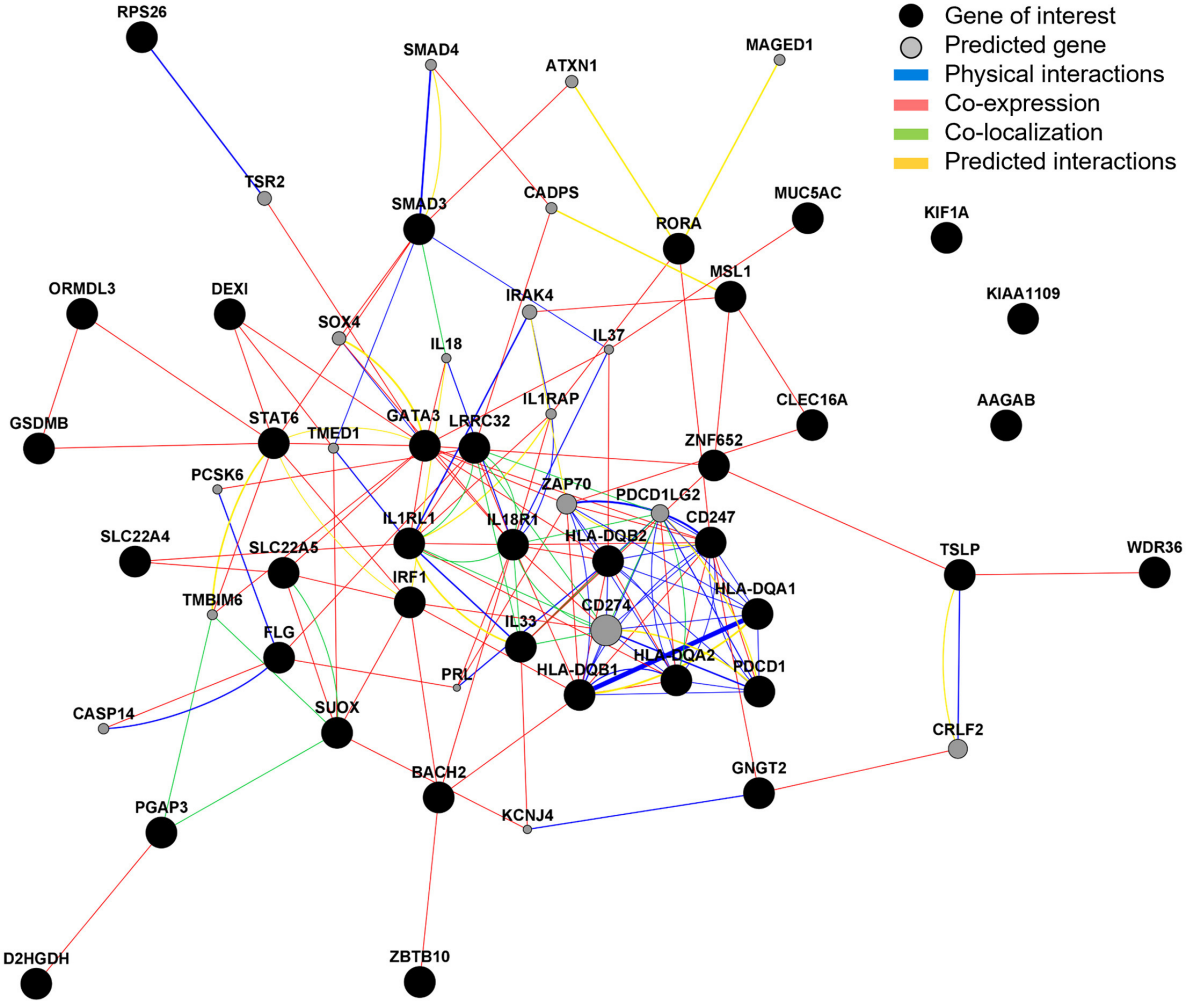
Gene interaction analysis. GeneMANIA was used to explore and predict gene interactions. Black circles represent inputted candidate genes and gray circles are predicted genes (selected to show top 20 with strongest predicted association). The area of the gray circle represents prediction scores and thickness of line represent interaction scores, with a larger area/thicker line representing stronger prediction/interaction.

Gene interaction analysis of the 37 candidate genes identified that the strongest physical interactions were observed between *HLA-DQA1* and *B1, SMAD3* and *SMAD4* (predicted gene), *CD247* and *ZAP70* (predicted gene), and *FLG* and *CASP14* (predicted gene). *IL33, LRRC32, IL18R1* and CD274 (predicted gene) were observed to have the greatest number of co-localized partners. *GATA3* and *IL18R1* were observed to have the greatest number of co-expressional partners. *AAGAB, KIF1A*, and *KIAA1109* showed no interaction between any other gene (Figure 8). The predicted genes with the strongest interactions were *CD274, IL37, IRAK4, PDCD1LG2*, and *ZAP70* (Supplementary Table 7).

### Gene Drug Interaction and Clinical Trial Data Analyses Identifies 14 Genes Targeted by Licensed Therapeutics and 5 Genes Under Clinical Trials for Asthma

Of the 37 candidate causal genes and five predicted genes, DGIdb identified that 14 of the genes have known interactions with currently available therapeutics, with highest scoring genes being, *SLC22A5* (Carnitine, 63.79), *MUC5AC* (Ensituximab, 31.9), and *CD274* (Avelumab, 23.92). Novel inhibitory antibodies under Phase II/III/IV clinical trials have been identified as potential novel therapeutics for asthma using https://clinicaltrials.gov, for 5 of the highlighted genes [*GATA3, IL1RL1, IL33, SMAD3*, and *TSLP* (Table 7)].

**Table 7:** Known drug interactions and molecules currently in clinical trials of 37 candidate causal genes and five predicted genes.

**Gene**	**Drug**	**Interaction type/company/disorder**	**PMIDs**	**Interaction score**	**clinicaltrials.gov**
AAGAB	-	-	-	-	-
BACH2	-	-	-	-	-
CD247	MUROMO-B-CD3	-	2503348, 8861551	15.95	-
CD247	BLI-TUMOMAB	-	-	3.04	-
CD247	AZACITIDINE	-	15795105	1.25	-
CD274	AVELUMAB	Antibody (inhibitory) Clinical Trials	28472902, 26918451, 23724846	23.92	^*^
CD274	ATEZOLIZUMAB	Antibody (inhibitory) Clinical Trials	24403232, 24829850, 28472902, 26970723, 26918451, 26952546, 27412122	10.63	^*^
CD274	DURVALUMAB	Antibody (inhibitory) Clinical Trials	28472902, 29416316, 28717238, 28214651	7.97	^*^
CD274	LODAPOLIMAB	Antibody (inhibitory)	-	5.32	-
CD274	CX-072	Clinical Trials/ CytomX Therapeutics/Solid tumor	-	5.32	NCT03993379, NCT03013491, NCT04596150
CD274	BMS-936559	Antibody (inhibitory) Clinical trials/Bristol-Myers Squibb/cancer and NIAID/HIV	-	5.32	NCT01452334, NCT01455103, NCT02576457, NCT02028403
CD274	ENVAFOLIMAB	Antibody (inhibitory) Clinical Trial/ Tracon Pharmaceuticals Inc./ Undifferentiated Pleomorphic Sarcoma|Myxofibrosarcoma	-	5.32	NCT04480502
CD274	PIDILIZUMAB	Antibody (inhibitory) Clinical trial/ Northwestern University|Gateway for Cancer Research|National Cancer Institute (NCI)/ Lymphoma	-	2.66	NCT02530125
CD274	NIVOLUMAB	Antibody (inhibitory) Clinical Trials	26086854, 28472902, 26918451	2.48	^*^
CD274	PEMBROLIZUMAB	Antibody (inhibitory) Clinical Trials	25891174, 28472902, 27718847, 26918451, 26086854	2.17	^*^
CD274	M-7824	Clinical Trials	-	1.77	^*^
CLEC16A	-	-	-	-	-
D2HGDH	-	-	-	-	-
DEXI	-	-	-	-	-
FLG	-	-	-	-	-
GATA3	PEGASPARGASE	-	24141364	2.45	-
GATA3	SB010	Clinical Trial/ Sterna Biologicals GmbH & Co. KG/Asthma	-	-	NCT01743768
GATA3	SB011	Clinical Trial/ Sterna Biologicals GmbH & Co. KG/ Mild to Moderate Atopic Dermatitis	-	-	NCT02079688
GATA3	SB012	Clinical Trial/ Sterna Biologicals GmbH & Co. KG/Ulcerative Colitis	-	-	NCT02129439
GNGT2	-	-	-	-	-
GSDMB	-	-	-	-	-
HLA-DQA1	LUMIRACOXIB	-	20639878	7.97	-
HLA-DQA1	LAPATINIB	-	24687830, 21245432	2.28	-
HLA-DQA1	AZATHIOPRINE	-	25217962	1.28	-
HLA-DQA2	-	-	-	-	-
HLA-DQB1	LUMIRACOXIB	-	20639878	2.66	-
HLA-DQB1	BUCILLAMINE	-	-	2.66	-
HLA-DQB1	CLAVULANIC ACID	-	10535882, 30664875	2.28	-
HLA-DQB1	FLOXACILLIN	-	30664875	1.52	-
HLA-DQB2	-	-	-	-	-
IL18R1	-	-	-	-	-
IL1RL1	MSTT1041A	Clinical Trial/University of Leicester/COPD	-	-	NCT03615040
IL1RL1	GSK3772847	Clinical Trial/GSK/Moderately Severe Asthma			NCT03207243
IL33	ITEPEKIMAB	Clinical Trial/Regerenron/COPD	-	-	NCT04701983
IL33	MSTT1041A	Clinical Trial/ Hoffmann-La Roche/Severe Asthma	-	-	NCT02918019
IL33	MEDI3506	Clinical Trial/AstraZenica/Uncontrolled Moderate-Severe Asthma	-	-	NCT04570657
IL33	ANB020	Clinical Trial/University of Leicester/AnaptysBio/Asthma	-	-	NCT04256044
IL37	-	-	-	-	-
IRAK4	PF-06650833	Inhibitory Clinical Trial/Yale University/COVID-19 Clinical Trial/Pfizer/rheumatoid arthritis	-	3.36	NCT04575610, NCT02996500
IRAK4	KT-474	Small molecule degrader Clinical Trial/ Kymera Therapeutics Inc./Atopic Dermatitis, Hidradenitis Suppurativa	-	-	NCT04772885
IRAK4	CA-4948	Inhibitory Clinical Trial/Curis Inc./Acute Myelogenous Leukemia, Myelodysplastic Syndrome/ Relapsed Hematologic Malignancy, Refractory Hematologic Malignancy	-	-	NCT04278768, NCT03328078
IRF1	-	-	-	-	-
KIF1A	-	-	-	-	-
KIAA1109	-	-	-	-	-
LRRC32	-	-	-	-	-
MSL1	-	-	-	-	-
MUC5AC	ENSITUXIMAB	-	-	31.9	-
ORMDL3	-	-	-	-	-
PDCD1	CEMIPLIMAB	Antibody (inhibitory), inhibitor (inhibitory)	29863979, 29089720	9.11	-
PDCD1	SPARTALIZUMAB	Antibody (inhibitory)	-	9.11	-
PDCD1	TISLELIZUMAB	Antibody (inhibitory)	-	9.11	-
PDCD1	PIDILIZUMAB	Antibody (inhibitory)	-	6.83	-
PDCD1	AMP-224	Antibody (inhibitory)	-	4.56	-
PDCD1	MGA-012	-	-	4.56	-
PDCD1	BALSTILIMAB	-	-	4.56	-
PDCD1	SYM-021	-	-	4.56	-
PDCD1	SASANLIMAB	-	-	4.56	-
PDCD1	SINTILIMAB	-	-	4.56	-
PDCD1	DOSTARLIMAB	-	-	4.56	-
PDCD1	NIVOLUMAB	Inhibitor (inhibitory), antibody (inhibitory)	23289116	2.13	-
PDCD1	M-7824	-	-	1.52	-
PDCD1	PEMBROLIZUMAB	Antibody (inhibitory), antagonist (inhibitory), inhibitor (inhibitory)	25685857	1.04	-
PDCD1	Pembrolizumab/ Vibostolimab Coformulation (MK-7684A)	Clinical Trial/Merck Sharp & Dohme Corp./ Metastatic Non-Small Cell Lung Cancer	-	-	NCT04725188
PDCD1LG2	AMP-224	Antibody	-	15.95	-
PDCD1LG2	PEMBROLIZUMAB	-	28619999	2.9	-
PGAP3	-	-	-	-	-
RORA	CHOLESTEROL	Agonist (activating)	10592235, 17139284, 17016423	31.9	-
RORA	T091317	Agonist (activating)	-	2.66	-
RORA	MELATONIN	-	8702678, 12595746	1.77	-
RPS26	-	-	-	-	-
SLC22A4	IMATINIB	-	23127916, 22875622	2.81	-
SLC22A5	CARNITINE	-	21422191	63.79	-
SLC22A5	IMATINIB	-	28762371, 23127916	1.41	-
SMAD3	VACTOSERTIB	Clinical Trial/ MedPacto, Inc./Solid state tumors	-	-	NCT02160106
STAT6	CHEMBL1374370	-	-	5.32	-
STAT6	CHEMBL363332	-	-	2.66	-
SUOX	-	-	-	-	-
TSLP	MEDI9929	Clinical Trial/ MedImmune LLC/Severe Asthma, Atopic Dermatitis	-	-	NCT02698501, NCT02054130, NCT02512900
TSLP	MRx-4DP0004	Clinical Trial/ 4D pharma plc/Asthma & COVID-19	-	-	NCT03851250, NCT04363372
WDR36	-	-	-	-	-
ZAP70	TRIDOLGOSIR	-	17897956	21.26	
ZAP70	ALOISINE	Inhibitory		10.63	
ZBTB10	-	-	-	-	-
ZNF652	-	-	-	-	-

*Interaction scores for known drug interactions with genes highlighted by our signal to gene analysis from the Drug Gene Interaction Database (DGIdb) are listed. The score is a numeric representation of publication count and source count, the ratio of average known gene partners for all drugs to the known partners for the given drug, and the ratio of average known drug partners for all genes to the known partners for the given gene. In interaction score cut off value of >1.0 were selected. Genes with drugs in clinical trials are also listed, CD274 had over 2,000 entries in clinicaltrials.gov and therefore drugs which appeared on the clinical trials database but not the DGIdb have been shown in Supplementary Material*.

* *Too many clinical trials to list, see Supplementary Material*.

## Discussion

Asthma is a heterogenous disease in which both genetic and environmental factors contribute to susceptibility and progression (35). Severe asthma, characterized by uncontrolled symptoms, a burden of medication and frequent exacerbations, remains inadequately managed in many patients (2). The current study aimed to provide significant translation of 25 genetic signals identified as risk factors for the development of moderate to severe asthma to new biological insight using a broad range of approaches and datasets. The main findings are (i) moderate-severe asthma genetic signals drive a large number of inflammatory cell traits particularly eosinophil levels and are risk factors for related traits such as atopic dermatitis and other inflammatory diseases, (ii) our signal to gene pipeline identified 37 candidate causal genes, (iii) genes show enrichment in lung tissue and inflammatory cells illustrating both the role in inflammation and structural changes in the airways, (iv) 32 of the 37 genes had additional support for a role in asthma including some with differential expression in the airways and blood cells of severe patients, (v) genes show enrichment for pathways relevant to T cell function, interferon signaling, endoplasmic reticulum (ER) biology and others, (vi) gene interaction analyses identified predicted genes already known to be involved in asthma and finally, (vii) these genes and pathways show potential as targets for novel drug development or repurposing.

### PheWAS Identified Features of Asthma and Other Traits Driven by Moderate-Severe Asthma Signals

Our initial analysis focused on understanding the uniqueness of the moderate-severe asthma signals by testing for association with a large number other traits *via* PheWAS. This approach can identify novel associations with quantitative traits e.g., lung function and provide significant inference regarding the mechanisms underlying the original association. All signals showed associations with the asthma trait including the *MUC5AC* signal that was identified as a potential moderate-severe asthma signal originally (7) and blood/immune cell trait associations were very prominent. Recently, this *MUC5AC* signal has been identified as potentially more relevant to adult onset asthma (36), a phenotype that shares less overlap with atopic comorbidities than childhood onset asthma. This may explain, at least in part, the differential profile compared to many of the other signals which showed associations with e.g., atopic dermatitis.

Interestingly, some signals showed trait specificity whilst others had broader trait associations. Of particular note are signals rs9273410 and rs776111176 both corresponding to *HLA-DQA1/A2/B1/B2* candidate genes, which had the widest disease associations including Hay fever, type 1 diabetes, inflammatory bowel disease, ulcerative colitis and psoriasis. The gene association for these results were confirmed in DAVID pathway and gene enrichment analysis where the *HLA-DQA1/A2/B1/B2* gene cluster was present in ~74% of diseases and pathways. Interestingly, PheWAS analysis for these signals showed a protective effect for some auto-immune diseases. Previous studies of signals in this region have confirmed inverse disease dependence risk between asthma and autoimmune disease, for example signal rs2395185, the asthma risk allele T was shown to be protective for ulcerative colitis (37, 38). These differences in trait profiles across diseases may offer insight into disease pathophysiology for this gene cluster.

Another notable association with the majority of signals was the association with blood eosinophils levels, with eosinophils known to be an effector cell in asthma and linked to severe asthma pathology (39). Indeed, current treatments such as Mepolizumab, Benralizumab, and Reslizumab reduce exacerbations through blocking IL5/IL5 signaling and reducing eosinophil numbers (40–43).

Interestingly, signal rs2941522 corresponding to candidate genes *ORMDL3, GSDMB, PGAP3*, and *MSL1* had the strongest association with neutrophil counts. This observation may point to a particular pathology for carriers of this variant and a role in non-eosinophilic or neutrophilic asthma (44, 45). Multiple GWAS and functional studies relate asthma to *ORMDL3* and *GSDMB* (46, 47) however to our knowledge no studies have been published investigating this variant or linked genes with regards to neutrophils in asthma.

Signals, rs12479210, rs144829310, and rs1986009 had the strongest eosinophilic associations, corresponding to *IL1RL1, IL18R1, SLC22A4/5*, and *IL33* candidate causal genes. *IL18R1* and *IL1RL1* have been shown in many studies to have variants associated with asthma as well as having altered expression profiles in asthmatic subjects (3, 28). Predictive interaction analysis revealed strong physical interactions between *IL1RL1* and *TMED1* which is unsurprising as both are related to *IL33* signaling (48) which has been extensively researched in the context of asthma (49).

### Signal to Gene Analyses Identifies 37 Plausible Candidate Causal Genes

The complexity of signals identified through GWAS highlight the importance of considering relevant tissue compartments when determining signal to gene associations. For many of the signals, we identified multiple plausible candidate causal genes to a single signal, with different genes acting as eQTLs in different cells and tissue types. This is potentially as anticipated as it is feasible that a signal may influence multiple genes and pathways. For example, considering signal rs12479210, we observe an eQTL for *IL1RL1* in lung tissue but not in a T-cell population, where *IL18R1* is the observed eQTL. This strongly suggests that a single signal may drive the differential expression of multiple genes and contribute to asthma mechanisms in multiple ways, the *IL1RL1* signal remains one of the most reproducible asthma signals potentially for this reason (50). Additionally, we highlight that when eQTLs are common across different tissue types, these may present dramatically different effects on gene expression. For example, when considering the above locus of association, we observe a positive eQTL for *IL1RL1* in whole blood (B = 0.1), but a negative eQTL effect in whole lung (B = −0.372). This emphasizes the need for further analysis on these identified eQTLs in order to unpick the relationship between the eQTLs and asthma relevant phenotypes (51). The 37 identified candidate causal genes broadly mapped to; barrier formation/defense, cell death, DNA/RNA binding, membrane binding and transport, metabolic processes, and signal transduction. Importantly, independent biological studies of asthma have identified a potential role for several of these pathways e.g., epithelial barrier/defense (52) and DNA/RNA binding (53), however more novel mechanisms identified require further investigation.

### Gene to Asthma Analyses Provide Significant Support for a Role of Candidate Causal Genes in the Structural Changes in the Airways and Inflammation in Asthma

Structural remodeling is a major component of asthma pathophysiology resulting in the narrowing of the airways. Changes comprise of loss of ciliated cells, goblet cell metaplasia, increased sputum production, basement membrane thickening and smooth muscle cell hypertrophy, and hyperplasia leading to an overall decline in lung function (54–56). Airway remodeling is thought to occur in response predominantly to chronic inflammation, which is supported by studies showing that steroid treatment in asthmatic patients both reduces airway inflammation and has a positive effect on airway remodeling (57, 58). The strong association of these signals with inflammatory traits and a candidate causal genes list with genes predominantly involved in signal transduction indicates these signals may contribute to airway remodeling.

Immune cell infiltration of the lung epithelium is a magnifier of asthmatic inflammation (59). *STAT6* expression was increased in the blood cells of asthmatic patients compared to controls in the dataset analyzed in this study. It is a transcription activator and has been linked to goblet cell metaplasia and an increase in MUC5AC production (60). The MUC5AC protein is a secreted gel forming mucin and its protein levels have also been shown to be increased in asthmatic airway mucus (61). Furthermore, in the airway epithelial dataset analyzed, *MUC5AC* showed a significant increase in gene expression in patients with severe asthma. Indeed, IL-13, an inflammatory cytokine highly associated with asthma induced activation of STAT6 has been shown to increase mucin expression and mucus metaplasia in both airway epithelial cells and submucosal glands in mice and has been linked to goblet cell hyper/metaplasia in humans (62). Both BACH2 and RORA are also DNA binding proteins, however in blood cells, their expression values were significantly lower in asthmatics. RORA has been shown to play a role in lung development (63) and childhood asthma (64) and studies in mice have shown BACH2 to repress T-cell cytokine production (65, 66) indicating lower levels of this protein in asthma may contribute to unregulated inflammation. Together, these findings suggest that imbalances in transcriptional regulators in inflammatory cells may, in part, contribute to asthma pathology possibly *via* downstream processes which affect multiple cells types including the airway epithelium. However, some caution is required as these expression datasets were from mixed cell populations, so we cannot exclude the influence of different cell populations between groups driving some mRNA level differences. Furthermore, the signal for the *MUC5AC* candidate gene, rs11603634, associated with the asthma, eosinophils and platelets traits only, indicating a role specific to severe/eosinophilic asthma that is potentially not driven by allergic mechanisms due to the lack of association with related traits in PheWAS.

Eosinophils, mast cells and T-helper (Th) cells are the key producers of inflammatory cytokines, IL-4, IL-5, and IL-13, associated with Th2 asthma (59, 67). From the signal association with eosinophils in the PheWAS analysis, it suggests inflammation plays a key role in asthma pathogenesis. Indeed, more than half of the 37 candidate causal genes were found, through gene function and pathway analyses, to be involved in signaling pathways related to inflammatory cytokines. Some genes such as *TSLP, IL-33* and its receptor *IL1RL1* have well-established asthma inflammatory roles (51, 68). *IL1RL1*, showed increased expression in both airway epithelium and blood of asthmatic patients compared to controls. Furthermore, inflammatory pathology is supported by the presence of *GATA3* and *STAT6* in asthma and inflammatory gene clusters in the pathway analysis. Both of these transcription factors have been shown to be vital for Th2 cell activation in asthma (69). Expression of *IL18R1*, a receptor for IL-18, was also increased in asthmatic airway epithelium. IL-18 has been shown to regulate both Th1 and Th2 immune responses (70, 71) through INFγ and IL-12 (72) and induce IgE production *via* IL-4 and STAT6 (73). Additionally, for *IL1RL1* and *IL18R1* colocalization analysis revealed strong association with both lung tissue and blood/immune cells indicating infiltration into the lung.

### Gene Functional Annotation, Pathway, and Gene Interaction Analysis Provides Insight Into Asthma Mechanisms Including Prediction of Other Asthma Related Genes

Functional and pathway analysis has revealed that the 37 candidate genes are involved in predominantly transcriptional regulation, membrane interaction and cytokine signaling, indicating that these processes may be disrupted or imbalanced in patients with asthma. A strong *HLA-DQA1/A2/B1/B2* gene cluster has emerged involving in a broad range of processes including auto-immune disease, viral and bacterial infection, antigen presentation and ER/Golgi/vesicle membrane interaction. If the gene cluster is broadened to include *CD247*, then combined these five genes are present in over 75% pathway and GO terms and processes. The *HLA* genes are part of a class of major histocompatibility complex (MHC) molecules which present exogenous allergen on airway dendritic cells and interact with CD247, part of the T-cell antigen receptor complex, to achieve T-cell activation (74, 75). From the blood dataset utilized in this study, both *CD247* and *HLA-DQA1/A2* gene expression were found to be decreased in asthmatic patients. Although studies in asthma are lacking, type 2 (non-insulin dependent) diabetics have shown chronic inflammation resulted in immunosuppression and CD247 downregulation (76). Seeing as chronic inflammation is also present in severe asthmatics, it is possible that a similar process is involved. Indeed, the signals, rs9273410 and rs776111176, for the *HLA-DQ* genes also had a strong risk association with diabetes albeit, type 1 (insulin-dependent) diabetes. Genetic variants in the *HLA* region have long been associated with asthma (77), however deconvolution of this region has been difficult.

Another feature which has appeared is the interaction/involvement of the candidate genes with the ER apparatus. The role of the ER, ER stress and the unfolded protein response (UPR) in asthma remains unclear. An increase in ER stress has been reported in asthma, and ER stress has also been shown in turn to increase mucin production, including MUC5AC, and airway remodeling (78, 79). In some studies, ORMDL3, an ER transmembrane protein, has been heavily implicated in asthma related ER stress (78, 79). *ORMDL3* gene expression was shown to be increased in asthmatic airway epithelium but decreased in blood compared to controls in the datasets used in this study. In mice, *ORMDL3* expression has been shown to be induced by allergen, IL-4 and IL-13 *via* STAT6 and in bronchial epithelial cells, overexpression of *ORMDL3* has been shown to trigger activating transcription factor 6 (ATF6), which has also been implicated in airway remodeling in asthma (80). However, the role of *ORMDL3* in ER driven asthma pathology remains to be resolved (81, 82).

### Analyses of 42 Genes Identified in the Current Study Provide Significant Opportunities for Drug Development and Or/Repurposing

Of the 37 candidate genes and five strongest predicted genes, drugs for *GATA3, IL33, IL1RL1, SMAD3*, and *TSLP* are either already in use or in clinical trials for asthma, clearly validating these genes from our pipeline as therapeutic targets. Drugs supported by genetic evidence are twice as likely to go through the drug development pipeline, be successful in Phases II and III and ultimately go into clinic (83). Drugs that target *MUC5AC* (ENSITUXIMAB) and *STAT6* (CHEMBL363332 and CHEMBL1374370) have emerged as potential avenues of future research *via* repurposing for severe asthma and show these two proteins are viable therapeutic targets. The *PDCD1* and *CD274* gene had the largest number of drug associations, albeit predominantly chemotherapy drugs. Although not a prominent gene to come out of this analysis, *PDCD1* has been shown in a small study to be increased in asthma patients after whole lung allergen challenge (84). However, caution must be taken when interpreting drug-gene interactions. For example, there are many drugs available, which may offer a starting point for targeting of the *HLA-DQA1/B1* genes such as azathioprine, which is an immunosuppressant already in use to treat rheumatoid arthritis, Crohn's disease and ulcerative colitis. However, in the blood cell dataset analysis, *HLA-DQA1* was decreased in asthmatics indicating a lack of gene expression is linked to risk and therefore inhibitory drugs at best would be ineffective and at worst intensify the asthma phenotype.

### A Move to Personalized Medicine

For any therapy to be truly effective in a disease such as asthma which is heterogenous, medicines need to be stratified based on evidence that that drug target and/or pathway is driving disease and genetic variants may help identify these individuals. The combination of variants may lead to a particular type of asthma such as IL33 high, which then may be amenable to therapy targeting IL33. Therefore, asthma studies which measure gene expression and also determine genotype offer the most comprehensive basis for understanding the effects of these signals and genes, as then risk allele, gene expression profile, and asthma phenotype can be bridged. For example, the rs11603634 signal, with candidate gene *MUC5AC*, is inherited in ~50% of the European population. The airway epithelium datasets analyzed in this study showed increased expression of MUC5AC and a brief analysis in the original GWAS paper has shown the individuals homozygous for the risk allele, A, show increased *MUC5AC* expression compared to homozygous non-risk allele, C, individuals (7). Together these results indicate *MUC5AC* is a strong candidate for inhibitor therapy particularly for individuals that carry the risk allele.

On the other hand, signal rs61816761 (candidate gene *FLG*) is inherited in ~2% of the European population and was highly specific in only associating with eczema/dermatitis in addition to asthma. This signal includes a loss of function variant and results from this study are consistent with the literature which indicate loss of function variants in the gene are associated with asthma in children with eczema (23). Therefore, therapy for these individuals would require restoring the loss of either FLG itself or of another molecule in its pathway but would probably only be effective in individuals with this variant. Prediction analysis in this study revealed novel physical interactions with *CASP14* and *PCSK6* and co-expressional interactions with *PRL, CASP14*, and *LRRC32*. These interactions may be compromised in subjects with this variant and are options for future exploration.

Therefore, any therapy, including those being investigated for repurposing in asthma, should be stratified and targeted to take into account the individual's genotype. There is previous proof of concept for this e.g., the prevention of asthma exacerbation by the IL4R antagonist, pitrakinra was shown to be effective in carriers of specific IL4R genotypes, although the specific mechanism is not known at this time (85).

## Conclusions

The results from this study replicate and importantly extend previous GWAS translational study findings. A study by Dong et al. which integrated eQTL data and GWAS summary data, using Sherlock Bayesian analysis, identified 11 candidate genes implicated in severe asthma of which four genes (*HLA-DQA1, GNGT2, STAT6*, and *SLC22A5*) overlap with the genes identified through our pipeline (86). Furthermore, a study by El-Husseini et al. which focused on druggable candidate genes identified through GWAS and eQTL analysis also showed overlap with the genes identified in this study notably *SLC22A4* and *SUOX* (87). Collectively, these results provide direct support for the role of 37 candidate causal genes in moderate to severe asthma pathological mechanisms and indirect evidence through interaction for an additional five genes. Overall, the findings demonstrate the contribution of altered airway structural cell and inflammatory cell mechanisms underlying asthma including novel inferences regarding genes not previously identified as potentially contributing to asthma. These genes and pathways can form the basis of novel drug development and/or drug repurposing with the aim to treat moderate to severe asthma where there is an unmet clinical need potentially in a stratified approach using genetics to guide therapy to the individual patient.

## Data Availability Statement

Publicly available datasets were analyzed in this study. This data can be found at: GEO GSE69683; https://www.ncbi.nlm.nih.gov/geo/query/acc.cgi?acc=GSE69683, GEO GSE43696; https://www.ncbi.nlm.nih.gov/geo/query/acc.cgi?acc=GSE43696.

## Author Contributions

IS conceived the study, contributed to the design, and contributed to writing the manuscript. SH, YG, and IA provided U-BIOPRED datasets and contributed to drafting the manuscript. MP and KR carried out the analysis and wrote the manuscript. All authors contributed to the article and approved the submitted version.

## Funding

The IS laboratory receives grant support from Asthma UK, British Lung Foundation, NIHR, MRC, GSK, NC3Rs, BBSRC, and Boehringer Ingelheim. The U-BIOPRED initiative was funded by Innovative Medicines Initiative (EU-IMI 115010). The Genotype-Tissue Expression (GTEx) Project was supported by the Common Fund of the Office of the Director of the National Institutes of Health, and by NCI, NHGRI, NHLBI, NIDA, NIMH, and NINDS. The data used for the analyses described in this manuscript were obtained from the GTEx Portal on 16/06/2020.

## Conflict of Interest

The authors declare that the research was conducted in the absence of any commercial or financial relationships that could be construed as a potential conflict of interest.

## Publisher's Note

All claims expressed in this article are solely those of the authors and do not necessarily represent those of their affiliated organizations, or those of the publisher, the editors and the reviewers. Any product that may be evaluated in this article, or claim that may be made by its manufacturer, is not guaranteed or endorsed by the publisher.
