# Supplementary material for: Translational Analysis of Moderate to Severe Asthma GWAS Signals Into Candidate Causal Genes and Their Functional, Tissue-Dependent and Disease-Related Associations
Source: Front Allergy. 2021 Oct 18;2:738741. doi: 10.3389/falgy.2021.738741 (PMC8974692; doi:10.3389/falgy.2021.738741)
Supplement: Supplementary file 1 [file Table_1.docx]

**SUPPLEMENTARY MATERIAL**

Translational analysis of moderate to severe asthma GWAS signals into candidate causal genes and their functional, tissue-dependent and disease-related associations

Michael A. Portelli^†1^, Kamini Rakkar^†1*^, Sile Hu ^2^, Yike Guo ^2^, Ian M. Adcock ^3^ on behalf of the U-BIOPRED Study Group & Ian Sayers^1^

^†^These authors have contributed equally to this work and share first authorship

^1^Centre for Respiratory Research, Translational Medical Sciences, School of Medicine, National Institute for Health Research Nottingham Biomedical Research Centre, Nottingham University Biodiscovery Institute, University of Nottingham, Nottingham, United Kingdom

^2^Data Science Institute, Imperial College London, London, United Kingdom

^3^The National Heart and Lung Institute, Imperial College, London, United Kingdom

***Correspondence:** Kamini Rakkar, [kamini.rakkar2@nottingham.ac.uk](mailto:kamini.rakkar2@nottingham.ac.uk)

**Keywords:** GWAS, causal genes, eQTL, SNP, moderate to severe asthma

Running Title: Asthma GWAS and causal genes

**INDEX**

| **Table/Figure** | **Title** | **Page No.** |
| --- | --- | --- |
| Table S1. | GeneATLAS PheWAS for each signal. | 3 |
| Table S2. | Protein Atlas data for tissue/cell enriched or enhanced gene and protein expression. | 24 |
| Table S3. | DAVID Functional Annotation Tool analysis of the Genetic Association Database of complex diseases and disorders (GAD). | 26 |
| Table S4. | Frequency of genes in GAD Terms. | 27 |
| Table S5. | DAVID Functional Annotation Tool analysis of GO Terms. | 28 |
| Table S6. | DAVID Functional Annotation Tool analysis of the KEGG/REACTOME Pathway | 29 |
| Table S7. | GeneMANIA predicted gene scores | 30 |
| Figure S1. | mRNA expression of candidate causal genes in bronchial epithelial cells taken from patients with asthma and controls | 31 |
| Figure S2. | mRNA expression of candidate causal genes in blood taken from patients with asthma and control subjects. | 32 |

**Supplementary Table S1. GeneATLAS PheWAS for each signal****.**

Proxies were used for the following signals which were not present in the database: rs61816761 (rs61816766, R^2^ = 0.50), rs367983479 (rs1504215, R^2^ = 0.85), rs71266076 (rs7824993, R^2^ = 0.81), rs7305461 (rs1131017, R^2^ = 0.75), rs112502960 (rs62076439, R^2^ = 1.0) and rs61840192 (rs1031163, R^2^ = 1.0). Signals rs776111176 and rs560026225 (no proxies available) were not found and have not been displayed.

| **rs7523907** | | | | |
| --- | --- | --- | --- | --- |
| **Trait** | **Beta** | **p-value** | **Odds Ratio** | **Effect Allele** |
| Eosinophill percentage | 0.0240 | 2.34E-14 | - | T |
| asthma | 0.0047 | 9.28E-14 | 1.05 | T |
| allergy/hypersensitivity/anaphylaxis | 0.0033 | 8.50E-10 | 1.05 | T |
| hayfever/allergic rhinitis | 0.0027 | 9.07E-09 | 1.05 | T |
| Eosinophill count | 0.0013 | 1.67E-08 | - | T |
| J40-J47 Chronic lower respiratory diseases | 0.0024 | 1.62E-05 | 1.03 | T |
| J45 Asthma | 0.0021 | 1.95E-05 | 1.04 | T |
| **rs12479210** | | | | |
| **Trait** | **Beta** | **p-value** | **Odds Ratio** | **Effect Allele** |
| Eosinophill percentage | 0.0962 | 4.05E-197 | - | T |
| Eosinophill count | 0.0066 | 8.19E-180 | - | T |
| asthma | 0.0104 | 4.97E-58 | 1.11 | T |
| J45 Asthma | 0.0052 | 3.99E-25 | 1.09 | T |
| J40-J47 Chronic lower respiratory diseases | 0.0057 | 9.06E-24 | 1.08 | T |
| hayfever/allergic rhinitis | 0.0044 | 4.89E-20 | 1.09 | T |
| allergy/hypersensitivity/anaphylaxis | 0.0045 | 2.16E-16 | 1.07 | T |
| Neutrophill percentage | -0.1128 | 4.91E-13 | - | T |
| J33 Nasal polyp | 0.0013 | 1.88E-11 | 1.18 | T |
| Mean sphered cell volume | 0.0402 | 4.54E-06 | - | T |
| Ease of skin tanning | -0.0078 | 6.54E-06 | - | T |
| Mean corpuscular volume | 0.0296 | 5.15E-05 | - | T |
| emphysema/chronic bronchitis | 0.0012 | 5.57E-05 | 1.06 | T |
| **rs34290285** | | | | |
| **Trait** | **Beta** | **p-value** | **Odds Ratio** | **Effect Allele** |
| Eosinophill percentage | -0.0767 | 1.37E-101 | - | A |
| Eosinophill count | -0.0053 | 1.53E-95 | - | A |
| asthma | -0.0107 | 2.06E-49 | 0.90 | A |
| J45 Asthma | -0.0059 | 1.56E-25 | 0.91 | A |
| J40-J47 Chronic lower respiratory diseases | -0.0064 | 3.97E-24 | 0.92 | A |
| hayfever/allergic rhinitis | -0.0047 | 1.08E-18 | 0.92 | A |
| allergy/hypersensitivity/anaphylaxis | -0.0049 | 1.32E-15 | 0.93 | A |
| Lymphocyte percentage | 0.0733 | 9.26E-07 | - | A |
| eczema/dermatitis | -0.0017 | 5.32E-06 | 0.93 | A |
| **rs1837253** | | | | |
| **Trait** | **Beta** | **p-value** | **Odds Ratio** | **Effect Allele** |
| Eosinophill percentage | 0.0592 | 3.04E-62 | - | C |
| Eosinophill count | 0.0043 | 4.69E-62 | - | C |
| asthma | 0.0110 | 6.85E-53 | 1.11 | C |
| J33 Nasal polyp | 0.0023 | 4.55E-28 | 1.34 | C |
| J45 Asthma | 0.0058 | 2.82E-25 | 1.10 | C |
| J40-J47 Chronic lower respiratory diseases | 0.0062 | 1.81E-23 | 1.09 | C |
| hayfever/allergic rhinitis | 0.0049 | 3.81E-20 | 1.10 | C |
| allergy/hypersensitivity/anaphylaxis | 0.0054 | 1.68E-18 | 1.08 | C |
| J30-J39 Other diseases of upper respiratory tract | 0.0024 | 4.18E-08 | 1.07 | C |
| Number of self-reported non-cancer illnesses | 0.0216 | 7.43E-08 | - | C |
| nasal/sinus disorder | 0.0014 | 1.49E-06 | 1.10 | C |
| **rs1438673** | | |  |  |
| **Trait** | **Beta** | **p-value** | **Odds Ratio** | **Effect Allele** |
| Eosinophill count | -0.0038 | 3.60E-65 | - | T |
| Eosinophill percentage | -0.0526 | 5.94E-64 | - | T |
| asthma | -0.0074 | 6.25E-32 | 0.93 | T |
| hayfever/allergic rhinitis | -0.0043 | 4.31E-20 | 0.92 | T |
| J45 Asthma | -0.0044 | 2.66E-19 | 0.93 | T |
| allergy/hypersensitivity/anaphylaxis | -0.0043 | 4.27E-16 | 0.94 | T |
| J40-J47 Chronic lower respiratory diseases | -0.0039 | 7.12E-13 | 0.95 | T |
| J33 Nasal polyp | -0.0012 | 7.20E-11 | 0.86 | T |
| Monocyte percentage | 0.0184 | 9.85E-07 | - | T |
| **rs3749833** | | |  |  |
| **Trait** | **Beta** | **p-value** | **Odds Ratio** | **Effect Allele** |
| Mean platelet (thrombocyte) volume | -0.0285 | 7.72E-57 | - | C |
| Eosinophill count | 0.0036 | 5.38E-46 | - | C |
| Eosinophill percentage | 0.0436 | 8.07E-35 | - | C |
| asthma | 0.0084 | 8.54E-32 | 1.09 | C |
| Neutrophill count | 0.0267 | 5.87E-21 | - | C |
| Lymphocyte percentage | -0.1356 | 4.37E-20 | - | C |
| Standing height | -0.0860 | 1.25E-19 | - | C |
| White blood cell (leukocyte) count | 0.0319 | 1.28E-19 | - | C |
| Platelet count | 0.8731 | 2.52E-17 | - | C |
| Comparative height size at age 10 | -0.0089 | 5.39E-12 | - | C |
| J40-J47 Chronic lower respiratory diseases | 0.0041 | 3.58E-11 | 1.06 | C |
| J45 Asthma | 0.0036 | 1.86E-10 | 1.06 | C |
| Leg predicted mass (right) | -0.0125 | 3.48E-09 | - | C |
| Leg fat-free mass (right) | -0.0133 | 3.83E-09 | - | C |
| Leg fat-free mass (left) | -0.0130 | 1.08E-08 | - | C |
| Leg predicted mass (left) | -0.0121 | 1.21E-08 | - | C |
| Whole body water mass | -0.0467 | 2.34E-08 | - | C |
| M72 Fibroblastic disorders | 0.0013 | 2.42E-08 | 1.14 | C |
| Neutrophill percentage | 0.0943 | 4.46E-08 | - | C |
| Whole body fat-free mass | -0.0618 | 5.07E-08 | - | C |
| Basal metabolic rate | -8.2019 | 1.05E-07 | - | C |
| Sitting height | -0.0337 | 1.29E-07 | - | C |
| Number of self-reported non-cancer illnesses | 0.0209 | 1.64E-07 | - | C |
| J33 Nasal polyp | 0.0011 | 1.87E-07 | 1.15 | C |
| Trunk fat-free mass | -0.0300 | 2.44E-07 | - | C |
| Trunk predicted mass | -0.0286 | 2.84E-07 | - | C |
| Weight | -0.1211 | 5.03E-06 | - | C |
| Weight | -0.1207 | 6.26E-06 | - | C |
| Monocyte count | 0.0014 | 7.38E-06 | - | C |
| Mean corpuscular haemoglobin | -0.0141 | 1.55E-05 | - | C |
| Basophill count | 0.0004 | 1.61E-05 | - | C |
| Arm fat-free mass (left) | -0.0037 | 1.99E-05 | - | C |
| Arm predicted mass (left) | -0.0035 | 2.05E-05 | - | C |
| I10 Essential (primary) hypertension | 0.0035 | 2.58E-05 | 1.02 | C |
| Hand grip strength (right) | -0.0659 | 3.44E-05 | - | C |
| I10-I15 Hypertensive diseases | 0.0034 | 3.90E-05 | 1.02 | C |
| Arm fat-free mass (right) | -0.0033 | 5.69E-05 | - | C |
| **rs1986009** | | |  |  |
| **Trait** | **Beta** | **p-value** | **Odds Ratio** | **Effect Allele** |
| Eosinophill percentage | 0.1132 | 1.06E-176 | - | A |
| Eosinophill count | 0.0080 | 6.13E-169 | - | A |
| asthma | 0.0094 | 6.50E-31 | 1.10 | A |
| J45 Asthma | 0.0051 | 1.14E-15 | 1.09 | A |
| Neutrophill percentage | -0.1557 | 1.25E-15 | - | A |
| J40-J47 Chronic lower respiratory diseases | 0.0052 | 2.16E-13 | 1.07 | A |
| Lymphocyte count | 0.0089 | 2.37E-10 | - | A |
| Standing height | -0.0642 | 1.99E-09 | - | A |
| Mean platelet (thrombocyte) volume | -0.0111 | 4.22E-08 | - | A |
| psoriasis | -0.0014 | 2.96E-07 | 0.88 | A |
| eczema/dermatitis | 0.0021 | 9.63E-07 | 1.09 | A |
| White blood cell (leukocyte) count | 0.0183 | 4.37E-06 | - | A |
| Immature reticulocyte fraction | -0.0006 | 3.51E-05 | - | A |
| thyroid problem (not cancer) | -0.0024 | 4.81E-05 | 0.96 | A |
| **rs9273410** | | |  |  |
| **Trait** | **Beta** | **p-value** | **Odds Ratio** | **Effect Allele** |
| malabsorption/coeliac disease | 0.0061 | < 1E-300 | 4.13 | A |
| K90 Intestinal malabsorption | 0.0059 | < 1E-300 | 3.24 | A |
| E10 Insulin-dependent diabetes mellitus | 0.0034 | 7.69E-77 | 1.65 | A |
| Asthma | 0.0124 | 7.55E-76 | 1.13 | A |
| thyroid problem (not cancer) | 0.0074 | 6.26E-52 | 1.14 | A |
| K90-K93 Other diseases of the digestive system | 0.0057 | 1.44E-49 | 1.21 | A |
| hypothyroidism/myxoedema | 0.0057 | 4.87E-37 | 1.13 | A |
| J45 Asthma | 0.0065 | 3.55E-35 | 1.12 | A |
| E00-E07 Disorders of thyroid gland | 0.0052 | 6.56E-34 | 1.14 | A |
| E03 Other hypothyroidism | 0.0046 | 4.41E-32 | 1.15 | A |
| Mean reticulocyte volume | 0.1659 | 4.41E-32 | - | A |
| hyperthyroidism/thyrotoxicosis | 0.0022 | 2.42E-31 | 1.34 | A |
| J40-J47 Chronic lower respiratory diseases | 0.0066 | 1.59E-29 | 1.09 | A |
| Mean sphered cell volume | 0.0990 | 8.55E-27 | - | A |
| Red blood cell (erythrocyte) count | -0.0066 | 3.35E-25 | - | A |
| Diabetes | 0.0046 | 7.88E-25 | 1.11 | A |
| Lymphocyte count | -0.0117 | 1.33E-23 | - | A |
| Haemoglobin concentration | -0.0183 | 4.59E-23 | - | A |
| E10-E14 Diabetes mellitus | 0.0044 | 9.35E-23 | 1.10 | A |
| E16 Other disorders of pancreatic internal secretion | 0.0010 | 2.97E-20 | 1.55 | A |
| E15-E16 Other disorders of glucose regulation and pancreatic internal secretion | 0.0010 | 6.97E-20 | 1.54 | A |
| H36 Retinal disorders in diseases classified elsewhere | 0.0011 | 2.82E-19 | 1.42 | A |
| Mean platelet (thrombocyte) volume | 0.0146 | 2.55E-18 | - | A |
| Reticulocyte count | -0.0004 | 1.12E-17 | - | A |
| E05 Thyrotoxicosis [hyperthyroidism] | 0.0012 | 2.54E-15 | 1.30 | A |
| Number of self-reported non-cancer illnesses | 0.0285 | 2.34E-14 | - | A |
| Psoriasis | -0.0017 | 1.58E-13 | 0.86 | A |
| bowel problem | 0.0039 | 1.46E-12 | 1.06 | A |
| E14 Unspecified diabetes mellitus | 0.0012 | 1.93E-12 | 1.23 | A |
| Haematocrit percentage | -0.0379 | 3.54E-12 | - | A |
| White blood cell (leukocyte) count | -0.0229 | 4.66E-12 | - | A |
| High light scatter reticulocyte count | -0.0001 | 7.57E-12 | - | A |
| Reticulocyte percentage | -0.0070 | 9.00E-12 | - | A |
| Number of treatments/medications taken | 0.0357 | 1.28E-11 | - | A |
| Monocyte count | -0.0020 | 1.94E-11 | - | A |
| Mean corpuscular haemoglobin concentration | -0.0127 | 3.13E-11 | - | A |
| allergy/hypersensitivity/anaphylaxis | 0.0037 | 9.99E-11 | 1.06 | A |
| D64 Other anaemias | 0.0024 | 1.00E-10 | 1.09 | A |
| D60-D64 Aplastic and other anaemias | 0.0024 | 1.68E-10 | 1.09 | A |
| Mean corpuscular volume | 0.0492 | 3.92E-10 | - | A |
| Dermatology | -0.0025 | 8.88E-09 | 0.94 | A |
| E11 Non-insulin-dependent diabetes mellitus | 0.0025 | 1.03E-08 | 1.06 | A |
| High light scatter reticulocyte percentage | -0.0021 | 3.69E-08 | - | A |
| hayfever/allergic rhinitis | 0.0027 | 4.07E-08 | 1.05 | A |
| K51 Ulcerative colitis | -0.0011 | 4.51E-08 | 0.87 | A |
| Lymphocyte percentage | -0.0755 | 4.85E-08 | - | A |
| Red blood cell (erythrocyte) distribution width | 0.0093 | 1.46E-07 | - | A |
| Neutrophill percentage | 0.0844 | 1.69E-07 | - | A |
| H30-H36 Disorders of choroid and retina | 0.0015 | 5.95E-07 | 1.09 | A |
| connective tissue disorder | 0.0009 | 6.16E-07 | 1.15 | A |
| inflammatory bowel disease | -0.0010 | 1.14E-06 | 0.89 | A |
| Vasculitis | 0.0006 | 1.55E-06 | 1.22 | A |
| diabetic eye disease | 0.0005 | 2.83E-06 | 1.27 | A |
| D50-D53 Nutritional anaemias | 0.0015 | 2.99E-06 | 1.08 | A |
| bone disorder | 0.0015 | 3.30E-06 | 1.07 | A |
| L40 Psoriasis | -0.0008 | 3.73E-06 | 0.87 | A |
| L97 Ulcer of lower limb, not elsewhere classified | 0.0005 | 3.79E-06 | 1.23 | A |
| chronic/degenerative neurological problem | -0.0008 | 4.73E-06 | 0.88 | A |
| Anaemia | 0.0010 | 5.60E-06 | 1.11 | A |
| C44 Other malignant neoplasms of skin | -0.0018 | 6.36E-06 | 0.95 | A |
| C43-C44 Melanoma and other malignant neoplasms of skin | -0.0019 | 8.37E-06 | 0.95 | A |
| I77 Other disorders of arteries and arterioles | 0.0006 | 1.39E-05 | 1.18 | A |
| G35 Multiple sclerosis | -0.0005 | 1.85E-05 | 0.84 | A |
| D50 Iron deficiency anaemia | 0.0013 | 2.76E-05 | 1.07 | A |
| G35-G37 Demyelinating diseases of the central nervous system | -0.0006 | 3.73E-05 | 0.86 | A |
| J33 Nasal polyp | 0.0008 | 3.77E-05 | 1.11 | A |
| Platelet count | -0.4016 | 3.81E-05 | - | A |
| Haematology | 0.0011 | 5.01E-05 | 1.08 | A |
| M81 Osteoporosis without pathological fracture | 0.0011 | 5.11E-05 | 1.08 | A |
| **rs144829310** | | |  |  |
| **Trait** | **Beta** | **p-value** | **Odds Ratio** | **Effect Allele** |
| Eosinophill percentage | 0.1473 | 3.51E-268 | - | T |
| Eosinophill count | 0.0104 | 5.67E-257 | - | T |
| Asthma | 0.0144 | 2.41E-63 | 1.15 | T |
| J33 Nasal polyp | 0.0026 | 1.02E-26 | 1.41 | T |
| J45 Asthma | 0.0068 | 2.07E-24 | 1.12 | T |
| J40-J47 Chronic lower respiratory diseases | 0.0075 | 9.26E-24 | 1.11 | T |
| hayfever/allergic rhinitis | 0.0048 | 3.85E-14 | 1.09 | T |
| allergy/hypersensitivity/anaphylaxis | 0.0048 | 4.54E-11 | 1.07 | T |
| J30-J39 Other diseases of upper respiratory tract | 0.0032 | 7.18E-10 | 1.10 | T |
| Neutrophill percentage | -0.1201 | 4.75E-09 | - | T |
| nasal/sinus disorder | 0.0017 | 2.43E-07 | 1.13 | T |
| Ease of skin tanning | -0.0104 | 5.63E-06 | - | T |
| J32 Chronic sinusitis | 0.0009 | 2.44E-05 | 1.17 | T |
| **rs10905284** | | |  |  |
| **Trait** | **Beta** | **p-value** | **Odds Ratio** | **Effect Allele** |
| Eosinophill percentage | -0.0348 | 3.19E-28 | - | A |
| Eosinophill count | -0.0024 | 3.84E-25 | - | A |
| Lymphocyte percentage | 0.0857 | 8.46E-11 | - | A |
| asthma | -0.0037 | 1.16E-08 | 0.97 | A |
| Lymphocyte count | 0.0055 | 8.62E-07 | - | A |
| J45 Asthma | -0.0024 | 1.56E-06 | 0.96 | A |
| J40-J47 Chronic lower respiratory diseases | -0.0026 | 4.15E-06 | 0.97 | A |
| J33 Nasal polyp | -0.0008 | 1.46E-05 | 0.90 | A |
| **rs11603634** | | |  |  |
| **Trait** | **Beta** | **p-value** | **Odds Ratio** | **Effect Allele** |
| asthma | 0.0032 | 5.10E-07 | 1.03 | G |
| Eosinophill percentage | 0.0150 | 1.45E-06 | - | G |
| Eosinophill count | 0.0010 | 1.08E-05 | - | G |
| K80-K87 Disorders of gallbladder, biliary tract and pancreas | -0.0017 | 4.29E-05 | 0.96 | G |
| Platelet count | -0.3654 | 6.26E-05 | - | G |
| K80 Cholelithiasis | -0.0015 | 6.32E-05 | 0.96 | G |
| **rs7936312** | | |  |  |
| **Trait** | **Beta** | **p-value** | **Odds Ratio** | **Effect Allele** |
| Eosinophill percentage | 0.0640 | 1.63E-94 | - | T |
| Eosinophill count | 0.0045 | 5.44E-89 | - | T |
| asthma | 0.0104 | 1.86E-61 | 1.11 | T |
| hayfever/allergic rhinitis | 0.0049 | 6.70E-26 | 1.10 | T |
| allergy/hypersensitivity/anaphylaxis | 0.0053 | 6.21E-23 | 1.08 | T |
| J45 Asthma | 0.0047 | 1.63E-21 | 1.08 | T |
| J40-J47 Chronic lower respiratory diseases | 0.0048 | 3.48E-18 | 1.07 | T |
| dermatology | 0.0036 | 4.16E-18 | 1.09 | T |
| eczema/dermatitis | 0.0028 | 4.69E-18 | 1.12 | T |
| Neutrophill percentage | -0.0914 | 1.57E-09 | - | T |
| Number of self-reported non-cancer illnesses | 0.0210 | 2.27E-09 | - | T |
| Impedance of arm (left) | 0.3882 | 2.38E-09 | - | T |
| Impedance of arm (right) | 0.3543 | 1.52E-08 | - | T |
| inflammatory bowel disease | 0.0010 | 4.20E-08 | 1.13 | T |
| Impedance of whole body | 0.5418 | 7.21E-07 | - | T |
| K51 Ulcerative colitis | 0.0009 | 1.36E-06 | 1.12 | T |
| L30 Other dermatitis | 0.0006 | 5.23E-06 | 1.17 | T |
| Number of treatments/medications taken | 0.0212 | 1.77E-05 | - | T |
| **rs10519068** | | |  |  |
| **Trait** | **Beta** | **p-value** | **Odds Ratio** | **Effect Allele** |
| asthma | -0.0087 | 1.47E-20 | 0.92 | A |
| Eosinophill percentage | -0.0400 | 3.34E-18 | - | A |
| Eosinophill count | -0.0027 | 2.92E-16 | - | A |
| hayfever/allergic rhinitis | -0.0043 | 7.77E-10 | 0.92 | A |
| allergy/hypersensitivity/anaphylaxis | -0.0042 | 1.11E-07 | 0.94 | A |
| J45 Asthma | -0.0033 | 6.11E-06 | 0.95 | A |
| **rs72743461** | | |  |  |
| **Trait** | **Beta** | **p-value** | **Odds Ratio** | **Effect Allele** |
| asthma | 0.0110 | 3.53E-50 | 1.11 | A |
| Eosinophill percentage | 0.0492 | 1.27E-41 | - | A |
| Eosinophill count | 0.0033 | 2.80E-36 | - | A |
| J45 Asthma | 0.0054 | 1.26E-20 | 1.10 | A |
| J40-J47 Chronic lower respiratory diseases | 0.0058 | 2.54E-19 | 1.08 | A |
| hayfever/allergic rhinitis | 0.0041 | 1.35E-13 | 1.08 | A |
| allergy/hypersensitivity/anaphylaxis | 0.0042 | 2.26E-11 | 1.06 | A |
| Lymphocyte percentage | -0.0831 | 4.69E-08 | - | A |
| I25 Chronic ischaemic heart disease | -0.0029 | 2.05E-07 | 0.95 | A |
| heart attack/myocardial infarction | -0.0016 | 4.19E-06 | 0.93 | A |
| Impedance of whole body | 0.5826 | 5.72E-06 | - | A |
| I20-I25 Ischaemic heart diseases | -0.0027 | 5.85E-06 | 0.96 | A |
| E11 Non-insulin-dependent diabetes mellitus | -0.0021 | 9.53E-06 | 0.95 | A |
| Lymphocyte count | -0.0056 | 1.15E-05 | - | A |
| Impedance of arm (right) | 0.3224 | 1.17E-05 | - | A |
| other respiratory problems | 0.0011 | 2.20E-05 | 1.10 | A |
| Impedance of arm (left) | 0.3200 | 2.79E-05 | - | A |
| E10-E14 Diabetes mellitus | -0.0020 | 5.08E-05 | 0.96 | A |
| **rs7203459** |  |  |  |  |
| **Trait** | **Beta** | **p-value** | **Odds Ratio** | **Effect Allele** |
| Eosinophill count | -0.0051 | 9.79E-86 | - | C |
| Eosinophill percentage | -0.0701 | 3.52E-84 | - | C |
| asthma | -0.0088 | 2.83E-33 | 0.92 | C |
| J45 Asthma | -0.0046 | 1.22E-15 | 0.93 | C |
| J40-J47 Chronic lower respiratory diseases | -0.0048 | 8.55E-14 | 0.94 | C |
| allergy/hypersensitivity/anaphylaxis | -0.0044 | 2.34E-12 | 0.94 | C |
| Sitting height | 0.0424 | 5.91E-11 | - | C |
| hayfever/allergic rhinitis | -0.0036 | 7.10E-11 | 0.94 | C |
| Lymphocyte count | -0.0077 | 1.01E-09 | - | C |
| Number of self-reported non-cancer illnesses | -0.0229 | 2.03E-08 | - | C |
| Hand grip strength (right) | 0.0889 | 4.26E-08 | - | C |
| Neutrophill percentage | 0.0936 | 1.01E-07 | - | C |
| Hand grip strength (left) | 0.0858 | 1.22E-07 | - | C |
| eczema/dermatitis | -0.0020 | 1.40E-07 | 0.92 | C |
| Number of operations, self-reported | -0.0164 | 2.08E-06 | - | C |
| Number of treatments/medications taken | -0.0253 | 1.08E-05 | - | C |
| White blood cell (leukocyte) count | -0.0155 | 1.57E-05 | - | C |
| **rs2941522** | | |  |  |
| **Trait** | **Beta** | **p-value** | **Odds Ratio** | **Effect Allele** |
| White blood cell (leukocyte) count | 0.0763 | 3.59E-133 | - | T |
| Neutrophill count | 0.0609 | 1.13E-129 | - | T |
| Asthma | 0.0101 | 9.93E-58 | 1.10 | T |
| Monocyte percentage | -0.0597 | 8.71E-57 | - | T |
| Neutrophill percentage | 0.1939 | 2.59E-37 | - | T |
| Lymphocyte count | 0.0125 | 1.92E-30 | - | T |
| Lymphocyte percentage | -0.1194 | 4.42E-20 | - | T |
| Basophill count | 0.0007 | 2.16E-19 | - | T |
| J45 Asthma | 0.0040 | 5.06E-16 | 1.07 | T |
| Mean reticulocyte volume | -0.0966 | 1.26E-13 | - | T |
| J40-J47 Chronic lower respiratory diseases | 0.0040 | 1.92E-13 | 1.06 | T |
| hayfever/allergic rhinitis | 0.0029 | 8.03E-10 | 1.06 | T |
| allergy/hypersensitivity/anaphylaxis | 0.0030 | 2.51E-08 | 1.04 | T |
| emphysema/chronic bronchitis | 0.0016 | 1.55E-07 | 1.08 | T |
| Oily fish intake | -0.0092 | 2.76E-07 | - | T |
| Platelet distribution width | 0.0042 | 1.13E-06 | - | T |
| Eosinophill percentage | -0.0145 | 3.02E-06 | - | T |
| bronchitis | 0.0008 | 3.18E-06 | 1.12 | T |
| Eosinophill count | 0.0010 | 3.18E-06 | - | T |
| Number of self-reported non-cancer illnesses | 0.0156 | 9.16E-06 | - | T |
| Platelet count | -0.3829 | 2.36E-05 | - | T |
| Fresh fruit intake | -0.0121 | 3.28E-05 | - | T |
| Mean sphered cell volume | -0.0354 | 3.29E-05 | - | T |
| Mean platelet (thrombocyte) volume | 0.0064 | 4.94E-05 | - | T |
| inflammatory bowel disease | -0.0008 | 5.98E-05 | 0.91 | T |
| Salt added to food | 0.0066 | 6.09E-05 | - | T |
| **rs703816** | | |  |  |
| **Trait** | **Beta** | **p-value** | **Odds Ratio** | **Effect Allele** |
| asthma | 0.0072 | 2.65E-29 | 1.07 | C |
| Mean platelet (thrombocyte) volume | 0.0136 | 1.93E-16 | - | C |
| J45 Asthma | 0.0036 | 2.73E-13 | 1.06 | C |
| Eosinophill count | 0.0016 | 1.80E-12 | - | C |
| Sitting height | -0.0400 | 2.29E-12 | - | C |
| migraine | -0.0025 | 2.44E-12 | 0.92 | C |
| Eosinophill percentage | 0.0211 | 2.09E-11 | - | C |
| Standing height | -0.0545 | 1.04E-10 | - | C |
| J40-J47 Chronic lower respiratory diseases | 0.0036 | 1.19E-10 | 1.05 | C |
| Platelet distribution width | 0.0053 | 1.06E-09 | - | C |
| neurology | -0.0030 | 1.01E-08 | 0.95 | C |
| High light scatter reticulocyte percentage | 0.0020 | 2.54E-08 | - | C |
| High light scatter reticulocyte count | 0.0001 | 7.42E-08 | - | C |
| hayfever/allergic rhinitis | 0.0023 | 1.21E-06 | 1.04 | C |
| allergy/hypersensitivity/anaphylaxis | 0.0026 | 2.13E-06 | 1.04 | C |
| Platelet count | -0.4346 | 2.91E-06 | - | C |
| Salt added to food | 0.0075 | 8.17E-06 | - | C |
| Trunk predicted mass | -0.0215 | 1.50E-05 | - | C |
| Trunk fat-free mass | -0.0221 | 1.96E-05 | - | C |
| Alcohol intake frequency. | -0.0117 | 4.42E-05 | - | C |
| Reticulocyte percentage | 0.0040 | 4.51E-05 | - | C |
| Arm fat percentage (left) | 0.0525 | 5.98E-05 | - | C |
| Arm fat percentage (right) | 0.0520 | 6.38E-05 | - | C |
| **rs61816761 (rs61816766 proxy, R^2^ = 0.50)** | | |  |  |
| **Trait** | **Beta** | **p-value** | **Odds Ratio** | **Effect Allele** |
| eczema/dermatitis | 0.0175 | 8.49E-77 | 2.02 | C |
| dermatology | 0.0173 | 5.81E-47 | 1.53 | C |
| asthma | 0.0163 | 3.86E-19 | 1.17 | C |
| Impedance of arm (right) | 1.5923 | 3.45E-18 | - | C |
| Impedance of arm (left) | 1.5021 | 2.86E-15 | - | C |
| Impedance of whole body | 1.7950 | 2.02E-08 | - | C |
| L30 Other dermatitis | 0.0019 | 2.58E-07 | 1.68 | C |
| J45 Asthma | 0.0072 | 4.36E-07 | 1.13 | C |
| Hand grip strength (right) | 0.1957 | 1.42E-06 | - | C |
| Hand grip strength (left) | 0.1851 | 5.05E-06 | - | C |
| J40-J47 Chronic lower respiratory diseases | 0.0071 | 7.27E-06 | 1.10 | C |
| L72 Follicular cysts of skin and subcutaneous tissue | -0.0035 | 4.15E-05 | 0.84 | C |
| **rs367983479 (rs1504215 proxy, R^2^ = 0.85)** | | |  |  |
| **Trait** | **Beta** | **p-value** | **Odds Ratio** | **Effect Allele** |
| Eosinophill count | -0.0043 | 7.64E-75 | - | A |
| Eosinophill percentage | -0.0528 | 1.70E-58 | - | A |
| Mean reticulocyte volume | 0.1673 | 2.27E-33 | - | A |
| Monocyte percentage | 0.0458 | 4.32E-31 | - | A |
| asthma | -0.0075 | 1.53E-29 | 0.93 | A |
| Mean sphered cell volume | 0.1008 | 2.37E-28 | - | A |
| thyroid problem (not cancer) | 0.0049 | 6.84E-24 | 1.09 | A |
| Mean corpuscular volume | 0.0744 | 9.55E-22 | - | A |
| hypothyroidism/myxoedema | 0.0042 | 5.79E-21 | 1.09 | A |
| White blood cell (leukocyte) count | -0.0289 | 1.14E-18 | - | A |
| Lymphocyte count | -0.0098 | 3.02E-17 | - | A |
| Mean corpuscular haemoglobin | 0.0255 | 6.18E-16 | - | A |
| J45 Asthma | -0.0041 | 4.31E-15 | 0.93 | A |
| E03 Other hypothyroidism | 0.0030 | 6.81E-15 | 1.09 | A |
| J40-J47 Chronic lower respiratory diseases | -0.0044 | 1.89E-14 | 0.94 | A |
| Red blood cell (erythrocyte) count | -0.0047 | 1.04E-13 | - | A |
| E00-E07 Disorders of thyroid gland | 0.0030 | 7.11E-13 | 1.08 | A |
| J33 Nasal polyp | -0.0013 | 1.31E-11 | 0.85 | A |
| Platelet crit | -0.0005 | 7.45E-11 | - | A |
| Neutrophill count | -0.0159 | 1.79E-09 | - | A |
| C44 Other malignant neoplasms of skin | -0.0023 | 6.01E-09 | 0.93 | A |
| C43-C44 Melanoma and other malignant neoplasms of skin | -0.0023 | 1.68E-08 | 0.94 | A |
| Platelet count | -0.4646 | 1.42E-06 | - | A |
| hayfever/allergic rhinitis | -0.0022 | 6.75E-06 | 0.96 | A |
| chronic obstructive airways disease/copd | -0.0005 | 2.32E-05 | 0.85 | A |
| **rs71266076 (rs7824993 proxy, R^2^ = 0.81)** | | |  |  |
| **Trait** | **Beta** | **p-value** | **Odds Ratio** | **Effect Allele** |
| asthma | -0.0068 | 1.83E-24 | 0.94 | G |
| Eosinophill percentage | -0.0250 | 1.81E-14 | - | G |
| Eosinophill count | -0.0017 | 2.03E-13 | - | G |
| J45 Asthma | -0.0035 | 6.84E-12 | 0.94 | G |
| J40-J47 Chronic lower respiratory diseases | -0.0036 | 5.96E-10 | 0.95 | G |
| Number of self-reported non-cancer illnesses | -0.0180 | 1.14E-06 | - | G |
| Number of operations, self-reported | -0.0133 | 2.17E-05 | - | G |
| Haemoglobin concentration | -0.0074 | 4.24E-05 | - | G |
| hayfever/allergic rhinitis | -0.0020 | 5.67E-05 | 0.96 | G |
| Haematocrit percentage | -0.0214 | 5.72E-05 | - | G |
| **rs7305461 (rs1131017 proxy, R^2^ = 0.75)** | | |  |  |
| **Trait** | **Beta** | **p-value** | **Odds Ratio** | **Effect Allele** |
| Eosinophill percentage | -0.0427 | 3.30E-42 | - | G |
| Eosinophill count | -0.0028 | 3.92E-34 | - | G |
| Asthma | -0.0061 | 1.18E-21 | 0.94 | G |
| Impedance of arm (left) | -0.5935 | 1.74E-19 | - | G |
| Arm predicted mass (left) | 0.0065 | 4.54E-19 | - | G |
| Impedance of whole body | -0.9859 | 4.70E-19 | - | G |
| Impedance of arm (right) | -0.5604 | 8.67E-19 | - | G |
| Arm fat-free mass (left) | 0.0068 | 2.23E-18 | - | G |
| Whole body water mass | 0.0627 | 3.19E-17 | - | G |
| Trunk fat-free mass | 0.0435 | 3.41E-17 | - | G |
| Trunk predicted mass | 0.0416 | 4.16E-17 | - | G |
| Whole body fat-free mass | 0.0840 | 7.30E-17 | - | G |
| Arm fat-free mass (right) | 0.0060 | 2.92E-16 | - | G |
| Arm predicted mass (right) | 0.0056 | 3.95E-16 | - | G |
| Basal metabolic rate | 11.0910 | 5.32E-16 | - | G |
| hypothyroidism/myxoedema | -0.0032 | 4.23E-14 | 0.93 | G |
| Leg fat-free mass (right) | 0.0148 | 1.30E-13 | - | G |
| Leg predicted mass (right) | 0.0139 | 1.34E-13 | - | G |
| Impedance of leg (left) | -0.3979 | 2.19E-13 | - | G |
| Leg fat-free mass (left) | 0.0147 | 2.82E-13 | - | G |
| Leg predicted mass (left) | 0.0137 | 3.61E-13 | - | G |
| Impedance of leg (right) | -0.3912 | 5.35E-13 | - | G |
| Lymphocyte count | 0.0079 | 7.93E-13 | - | G |
| J45 Asthma | -0.0033 | 3.01E-11 | 0.95 | G |
| Weight | 0.1574 | 3.04E-11 | - | G |
| J40-J47 Chronic lower respiratory diseases | -0.0036 | 1.22E-10 | 0.95 | G |
| Body mass index (BMI) | 0.0517 | 1.88E-10 | - | G |
| Lymphocyte percentage | 0.0827 | 2.82E-10 | - | G |
| Weight | 0.1480 | 3.16E-10 | - | G |
| thyroid problem (not cancer) | -0.0029 | 5.72E-10 | 0.95 | G |
| E03 Other hypothyroidism | -0.0022 | 2.09E-09 | 0.94 | G |
| Body mass index (BMI) | 0.0482 | 2.29E-09 | - | G |
| hayfever/allergic rhinitis | -0.0027 | 1.34E-08 | 0.95 | G |
| Smoking status | 0.0068 | 1.72E-07 | - | G |
| J33 Nasal polyp | -0.0010 | 1.74E-07 | 0.88 | G |
| Time spent watching television (TV) | 0.0155 | 4.27E-07 | - | G |
| Waist circumference | 0.1033 | 8.75E-07 | - | G |
| allergy/hypersensitivity/anaphylaxis | -0.0026 | 1.42E-06 | 0.96 | G |
| Processed meat intake | 0.0098 | 1.43E-06 | - | G |
| E00-E07 Disorders of thyroid gland | -0.0018 | 4.91E-06 | 0.95 | G |
| Red blood cell (erythrocyte) count | 0.0027 | 6.64E-06 | - | G |
| Arm fat mass (right) | 0.0047 | 8.14E-06 | - | G |
| Hypertension | 0.0036 | 1.08E-05 | 1.02 | G |
| Arm fat mass (left) | 0.0051 | 1.73E-05 | - | G |
| Leg fat mass (right) | 0.0115 | 2.20E-05 | - | G |
| Hip circumference | 0.0646 | 5.72E-05 | - | G |
| **rs112502960 (rs62076439, R^2^ = 1.0)** | | |  |  |
| **Trait** | **Beta** | **p-value** | **Odds Ratio** | **Effect Allele** |
| Impedance of leg (left) | -0.7662 | 4.66E-43 | - | T |
| Standing height | -0.1166 | 8.19E-42 | - | T |
| Impedance of whole body | -1.5375 | 8.50E-42 | - | T |
| Impedance of leg (right) | -0.7251 | 8.82E-39 | - | T |
| Eosinophill percentage | 0.0402 | 1.42E-35 | - | T |
| Eosinophill count | 0.0029 | 1.74E-35 | - | T |
| Impedance of arm (left) | -0.7696 | 4.36E-30 | - | T |
| Impedance of arm (right) | -0.7018 | 3.62E-27 | - | T |
| hypertension | 0.0083 | 2.45E-23 | 1.04 | T |
| asthma | 0.0060 | 1.01E-19 | 1.06 | T |
| Monocyte count | 0.0024 | 7.77E-17 | - | T |
| I10-I15 Hypertensive diseases | 0.0062 | 3.25E-16 | 1.04 | T |
| I10 Essential (primary) hypertension | 0.0062 | 3.48E-16 | 1.04 | T |
| Sitting height | -0.0459 | 2.88E-15 | - | T |
| Comparative body size at age 10 | 0.0094 | 3.37E-13 | - | T |
| Number of treatments/medications taken | 0.0343 | 2.61E-11 | - | T |
| Mean reticulocyte volume | 0.0901 | 2.90E-11 | - | T |
| J40-J47 Chronic lower respiratory diseases | 0.0038 | 4.01E-11 | 1.05 | T |
| I25 Chronic ischaemic heart disease | 0.0032 | 7.37E-11 | 1.06 | T |
| I20-I25 Ischaemic heart diseases | 0.0034 | 1.57E-10 | 1.05 | T |
| J45 Asthma | 0.0032 | 2.53E-10 | 1.06 | T |
| Monocyte percentage | 0.0239 | 9.11E-10 | - | T |
| angina | 0.0022 | 1.81E-09 | 1.07 | T |
| Trunk fat percentage | -0.0745 | 6.34E-09 | - | T |
| Platelet distribution width | -0.0051 | 1.26E-08 | - | T |
| Mean platelet (thrombocyte) volume | -0.0091 | 2.51E-08 | - | T |
| E78 Disorders of lipoprotein metabolism and other lipidaemias | 0.0031 | 9.05E-08 | 1.04 | T |
| Haematocrit percentage | 0.0279 | 1.21E-07 | - | T |
| I20 Angina pectoris | 0.0022 | 2.15E-07 | 1.05 | T |
| Number of self-reported non-cancer illnesses | 0.0179 | 1.01E-06 | - | T |
| heart/cardiac problem | 0.0026 | 1.54E-06 | 1.04 | T |
| Smoking status | 0.0062 | 3.54E-06 | - | T |
| Immature reticulocyte fraction | 0.0005 | 6.06E-06 | - | T |
| E70-E90 Metabolic disorders | 0.0029 | 6.11E-06 | 1.03 | T |
| Body fat percentage | -0.0495 | 8.77E-06 | - | T |
| Red blood cell (erythrocyte) count | 0.0027 | 1.23E-05 | - | T |
| Body mass index (BMI) | 0.0361 | 1.34E-05 | - | T |
| Body mass index (BMI) | 0.0363 | 1.39E-05 | - | T |
| Trunk fat mass | -0.0390 | 1.46E-05 | - | T |
| heart attack/myocardial infarction | 0.0014 | 1.57E-05 | 1.06 | T |
| High light scatter reticulocyte count | 0.0007 | 1.64E-05 | - | T |
| Mean time to correctly identify matches | 0.9348 | 2.09E-05 | - | T |
| Haemoglobin concentration | 0.0074 | 3.45E-05 | - | T |
| Time spent watching television (TV) | 0.0130 | 3.98E-05 | - | T |
| **rs61840192 (rs1031163, R^2^ = 1.0)** | | | |  |
| **Trait** | **Beta** | **p-value** | **Odds Ratio** | **Effect Allele** |
| Eosinophill percentage | -0.0650 | 5.66E-95 | - | T |
| Eosinophill count | -0.0046 | 3.74E-92 | - | T |
| asthma | -0.0096 | 6.85E-51 | 0.91 | T |
| J45 Asthma | -0.0061 | 4.40E-35 | 0.90 | T |
| J40-J47 Chronic lower respiratory diseases | -0.0062 | 5.17E-29 | 0.92 | T |
| J33 Nasal polyp | -0.0013 | 7.04E-12 | 0.85 | T |
| hayfever/allergic rhinitis | -0.0032 | 1.77E-11 | 0.94 | T |
| allergy/hypersensitivity/anaphylaxis | -0.0033 | 6.36E-10 | 0.95 | T |
| Number of self-reported non-cancer illnesses | -0.0165 | 3.55E-06 | - | T |
| J30-J39 Other diseases of upper respiratory tract | -0.0017 | 6.19E-06 | 0.95 | T |
| nasal/sinus disorder | -0.0011 | 2.28E-05 | 0.93 | T |
| **rs560026225 (rs72687036, R^2^ = 0.66)** |  |  |  |  |
| **Trait** | **Beta** | **p-value** | **Odds Ratio** | **Effect Allele** |
| Eosinophill percentage | 0.0346 | 1.42E-19 | - | G |
| asthma | 0.0066 | 2.50E-17 | 1.07 | G |
| Mean platelet (thrombocyte) volume | -0.0160 | 9.50E-17 | - | G |
| Eosinophill count | 0.0022 | 3.97E-15 | - | G |
| hayfever/allergic rhinitis | 0.0033 | 9.18E-09 | 1.06 | G |
| J45 Asthma | 0.0033 | 4.65E-08 | 1.06 | G |
| J40-J47 Chronic lower respiratory diseases | 0.0035 | 2.64E-07 | 1.05 | G |
| Mean reticulocyte volume | -0.0826 | 2.72E-07 | - | G |
| Lymphocyte count | -0.0069 | 3.03E-07 | - | G |
| Sitting height | 0.0343 | 7.20E-07 | - | G |
| Mean sphered cell volume | -0.0495 | 2.44E-06 | - | G |
| Lymphocyte percentage | -0.0713 | 9.08E-06 | - | G |
| Standing height | 0.0446 | 1.37E-05 | - | G |
| allergy/hypersensitivity/anaphylaxis | 0.0028 | 1.53E-05 | 1.04 | G |
| Number of treatments/medications taken | 0.0255 | 3.00E-05 | - | G |
| Mean corpuscular volume | -0.0353 | 5.45E-05 | - | G |
| K51 Ulcerative colitis | 0.0009 | 6.24E-05 | 1.12 | G |
| **rs776111176 (rs3997872, R^2^ = 0.82)** |  |  |  |  |
| **Trait** | **Beta** | **p-value** | **Odds Ratio** | **Effect Allele** |
| rheumatoid arthritis | 0.0077 | 4.64E-166 | 2.00 | A |
| M06 Other rheumatoid arthritis | 0.0068 | 3.38E-137 | 1.92 | A |
| White blood cell (leukocyte) count | 0.0870 | 1.25E-106 | - | A |
| Neutrophill count | 0.0680 | 3.37E-100 | - | A |
| M05 Seropositive rheumatoid arthritis | 0.0019 | 3.12E-84 | 4.01 | A |
| asthma | 0.0154 | 1.26E-81 | 1.16 | A |
| E10 Insulin-dependent diabetes mellitus | 0.0040 | 1.07E-76 | 1.83 | A |
| malabsorption/coeliac disease | -0.0031 | 5.90E-69 | 0.49 | A |
| hypothyroidism/myxoedema | 0.0093 | 6.62E-66 | 1.22 | A |
| Number of treatments/medications taken | 0.1040 | 6.38E-61 | - | A |
| K90 Intestinal malabsorption | -0.0031 | 7.77E-60 | 0.54 | A |
| Eosinophill count | 0.0044 | 2.77E-53 | - | A |
| thyroid problem (not cancer) | 0.0084 | 1.19E-46 | 1.17 | A |
| J45 Asthma | 0.0090 | 3.16E-46 | 1.16 | A |
| J40-J47 Chronic lower respiratory diseases | 0.0093 | 1.34E-40 | 1.13 | A |
| diabetes | 0.0070 | 5.03E-39 | 1.17 | A |
| E03 Other hypothyroidism | 0.0059 | 4.29E-36 | 1.19 | A |
| vasculitis | 0.0018 | 6.64E-36 | 1.87 | A |
| M05-M14 Inflammatory polyarthropathies | 0.0066 | 1.35E-33 | 1.16 | A |
| J33 Nasal polyp | 0.0027 | 1.95E-31 | 1.42 | A |
| E00-E07 Disorders of thyroid gland | 0.0059 | 4.97E-31 | 1.16 | A |
| E10-E14 Diabetes mellitus | 0.0062 | 5.88E-31 | 1.15 | A |
| Lymphocyte percentage | -0.1773 | 1.16E-26 | - | A |
| Number of self-reported non-cancer illnesses | 0.0476 | 2.61E-26 | - | A |
| H36 Retinal disorders in diseases classified elsewhere | 0.0015 | 1.88E-24 | 1.62 | A |
| Neutrophill percentage | 0.1964 | 3.45E-24 | - | A |
| Monocyte percentage | -0.0473 | 5.36E-23 | - | A |
| Mean sphered cell volume | -0.1066 | 6.22E-22 | - | A |
| M35 Other systemic involvement of connective tissue | 0.0016 | 8.50E-20 | 1.43 | A |
| joint disorder | 0.0078 | 1.21E-19 | 1.07 | A |
| Hand grip strength (right) | -0.1525 | 1.24E-17 | - | A |
| Waist circumference / Hip circumference | 0.0013 | 1.32E-16 | - | A |
| Lymphocyte count | 0.0114 | 4.95E-16 | - | A |
| G35 Multiple sclerosis | -0.0012 | 6.02E-16 | 0.68 | A |
| E11 Non-insulin-dependent diabetes mellitus | 0.0042 | 9.19E-16 | 1.10 | A |
| Reticulocyte count | 0.0004 | 1.02E-15 | - | A |
| chronic/degenerative neurological problem | -0.0017 | 1.49E-15 | 0.76 | A |
| Monocyte count | 0.0029 | 1.89E-15 | - | A |
| Reticulocyte percentage | 0.0097 | 2.87E-15 | - | A |
| Hand grip strength (left) | -0.1388 | 7.06E-15 | - | A |
| Eosinophill percentage | 0.0308 | 8.39E-15 | - | A |
| High light scatter reticulocyte count | 0.0002 | 2.08E-14 | - | A |
| G35-G37 Demyelinating diseases of the central nervous system | -0.0012 | 2.87E-14 | 0.71 | A |
| nasal/sinus disorder | 0.0023 | 1.14E-13 | 1.18 | A |
| High light scatter reticulocyte percentage | 0.0033 | 7.97E-13 | - | A |
| Mean corpuscular haemoglobin | -0.0265 | 4.04E-12 | - | A |
| Standing height | -0.0746 | 4.15E-12 | - | A |
| bowel problem | -0.0044 | 3.44E-11 | 0.93 | A |
| Sitting height | -0.0476 | 4.81E-11 | - | A |
| J30-J39 Other diseases of upper respiratory tract | 0.0032 | 5.81E-11 | 1.10 | A |
| connective tissue disorder | 0.0014 | 8.00E-11 | 1.24 | A |
| K51 Ulcerative colitis | -0.0015 | 8.84E-11 | 0.82 | A |
| ent disorder/not cancer | 0.0032 | 9.43E-11 | 1.10 | A |
| Platelet count | 0.7471 | 1.64E-10 | - | A |
| K90-K93 Other diseases of the digestive system | -0.0029 | 4.65E-10 | 0.91 | A |
| diabetic eye disease | 0.0007 | 1.86E-09 | 1.45 | A |
| Mean corpuscular volume | -0.0563 | 2.34E-09 | - | A |
| E16 Other disorders of pancreatic internal secretion | 0.0008 | 2.71E-09 | 1.40 | A |
| E15-E16 Other disorders of glucose regulation and pancreatic internal secretion | 0.0008 | 2.99E-09 | 1.40 | A |
| D06 Carcinoma in situ of cervix uteri | 0.0019 | 3.52E-09 | 1.27 | A |
| Trunk fat-free mass | -0.0384 | 4.33E-09 | - | A |
| Trunk predicted mass | -0.0364 | 6.57E-09 | - | A |
| psoriasis | -0.0016 | 2.04E-08 | 0.87 | A |
| inflammatory bowel disease | -0.0013 | 3.32E-08 | 0.85 | A |
| E14 Unspecified diabetes mellitus | 0.0011 | 3.50E-08 | 1.22 | A |
| Basophill count | 0.0005 | 7.59E-08 | - | A |
| M31 Other necrotising vasculopathies | 0.0005 | 8.07E-08 | 1.47 | A |
| sarcoidosis | -0.0006 | 1.01E-07 | 0.72 | A |
| K57 Diverticular disease of intestine | 0.0034 | 1.16E-07 | 1.06 | A |
| Whole body water mass | -0.0494 | 1.52E-07 | - | A |
| Platelet distribution width | 0.0057 | 1.85E-07 | - | A |
| eczema/dermatitis | 0.0022 | 1.88E-07 | 1.09 | A |
| Whole body fat-free mass | -0.0650 | 3.58E-07 | - | A |
| Platelet crit | 0.0005 | 3.70E-07 | - | A |
| H30-H36 Disorders of choroid and retina | 0.0017 | 6.88E-07 | 1.10 | A |
| Time spent watching television (TV) | 0.0190 | 9.06E-07 | - | A |
| hypertension | 0.0050 | 9.86E-07 | 1.03 | A |
| hyperthyroidism/thyrotoxicosis | -0.0011 | 1.05E-06 | 0.86 | A |
| Leg fat percentage (right) | 0.0549 | 4.54E-06 | - | A |
| Impedance of leg (left) | 0.3075 | 7.19E-06 | - | A |
| Impedance of whole body | 0.6220 | 8.52E-06 | - | A |
| Impedance of leg (right) | 0.3041 | 8.99E-06 | - | A |
| J20-J22 Other acute lower respiratory infections | 0.0016 | 1.06E-05 | 1.09 | A |
| Leg fat-free mass (right) | -0.0111 | 1.25E-05 | - | A |
| Leg predicted mass (right) | -0.0103 | 1.56E-05 | - | A |
| H00 Hordeolum and chalazion | 0.0008 | 1.69E-05 | 1.19 | A |
| J32 Chronic sinusitis | 0.0009 | 2.02E-05 | 1.16 | A |
| Leg fat percentage (left) | 0.0485 | 2.06E-05 | - | A |
| H00-H06 Disorders of eyelid, lacrimal system and orbit | 0.0017 | 2.20E-05 | 1.08 | A |
| Mean reticulocyte volume | -0.0710 | 2.55E-05 | - | A |
| Basal metabolic rate | -7.2895 | 2.64E-05 | - | A |
| J22 Unspecified acute lower respiratory infection | 0.0015 | 2.99E-05 | 1.09 | A |
| Body fat percentage | 0.0569 | 3.37E-05 | - | A |
| I10 Essential (primary) hypertension | 0.0038 | 3.98E-05 | 1.03 | A |
| Number of operations, self-reported | 0.0155 | 4.27E-05 | - | A |
| I10-I15 Hypertensive diseases | 0.0038 | 4.71E-05 | 1.03 | A |
| Arm fat-free mass (right) | -0.0037 | 5.36E-05 | - | A |

**Supplementary Table S2. Protein Atlas data for tissue/cell enriched or enhanced gene and protein expression.**

| **Gene** | **Tissue** | **Cell** |
| --- | --- | --- |
| AAGAB | None | None |
| BACH2 | Lymphoid | horizontal cells |
| CD247 | blood, lymphoid | T-cells |
| CLEC16A | None | early spermatids, late spermatids |
| D2HGDH | None | None |
| DEXI | skeletal muscle | None |
| FLG | oesophagus, skin, tongue | suprabasal keratinocytes |
| GATA3 | seminal vesicle, skin | syncythiotrophoblasts, extravillous trophoblasts, cytotrophoblasts |
| GNGT2 | blood, lymphoid, retina | cone photoreceptor cells, kupffer cells, macrophages |
| GSDMB | Intestine | enterocytes, pnaeth cells, mucus-secreting cells |
| HLA-DQA1 | blood, lymphoid | monocytes, macrophages, B-cells, Hofbauer cells |
| HLA-DQA2 | lung, lymphoid | macrophages,Hofbauer cells, B-cells, kupffer cells |
| HLA-DQB1 | Lymphoid | monocytes, macrophages, B-cells, Hofbauer cells |
| HLA-DQB2 | lymphoid, skin | macrophages, melanocytes, basal keratinocytes, suprabasal |
| IL18R1 | Lung | Granulocytes |
| IL1RL1 | kidney, lung, placenta | Granulocytes |
| IL33 | None | lto cells, endothelial cells, club cells, cilliated cells |
| IRF1 | Blood | none |
| KIF1A | Brain | Horizontal cells, Cone photoreceptor cells, Bipolar cells, Rod photoreceptor cells |
| KIAA1109 | Low | Rod photoreceptor cells |
| LRRC32 | None | extravillous trophoblasts, endothelial cells, smooth muscle cells, lto cells, fibroblasts, sertoli cells |
| MSL1 | None | none |
| MUC5AC | gallbladder, lung, stomach | club cells, ciliated cells, pancreatic endocrine cells |
| ORMDL3 | None | late spermatids, early spermatids |
| PDCD1 | blood, lymphoid tissue | T-cells |
| PGAP3 | None | none |
| RORA | Skin | cone photoreceptor cells, suprabasal keratinocytes |
| RPS26 | None | none |
| SLC22A4 | None | ciliated cells, erythroid cells |
| SLC22A5 | kidney, skeletal muscle | enterocytes |
| SMAD3 | Low | urothelial cells, glandular cells |
| STAT6 | None | None |
| SUOX | None | hepatocytes |
| TSLP | None | basal keratinocytes, club cells, early spermatids, basal glandular cells, suprabasal keratinocytes, late spermatids |
| WDR36 | None | basal keratinocytes |
| ZBTB10 | None | None |
| ZNF652 | None | None |

**Supplementary Table S3. DAVID Functional Annotation Tool analysis of the Genetic Association Database of complex diseases and disorders (GAD).** Candidate causal gene clusters and disease associations are listed from the GAD database. FE = fold enrichment.

| **Term** | **Genes** | **Count** | **%** | **FE** | **p-value** | **FDR** |
| --- | --- | --- | --- | --- | --- | --- |
| Asthma | FLG, IL33, GSDMB, SLC22A5, SMAD3, TSLP, GATA3, SUOX, MUC5AC, IL1RL1, IRF1, ORMDL3, STAT6, HLA-DQA2, IL18R1, HLA-DQA1, HLA-DQB1 | 17 | 45.95 | 14.53 | 1.78E-15 | 1.07E-12 |
| Diabetes Mellitus, Type 1 | KIAA1109, GSDMB, CLEC16A, PDCD1, SUOX, HLA-DQA2, BACH2, HLA-DQA1, HLA-DQB1 | 9 | 24.32 | 32.76 | 1.58E-10 | 4.76E-08 |
| Celiac disease | IL1RL1, KIAA1109, IRF1, CLEC16A, CD247, BACH2, IL18R1, HLA-DQA1, HLA-DQB1 | 9 | 24.32 | 22.97 | 2.72E-09 | 5.44E-07 |
| nasal polyposis | IL33, IL1RL1, WDR36, HLA-DQA1, HLA-DQB1 | 5 | 13.51 | 140.38 | 3.00E-08 | 4.30E-06 |
| obesity\|asthma | IL33, IL1RL1, GSDMB, TSLP, ORMDL3, IL18R1 | 6 | 16.22 | 60.47 | 3.57E-08 | 4.30E-06 |
| ulcerative colitis | SLC22A4, GSDMB, SLC22A5, ORMDL3, STAT6, HLA-DQA1, HLA-DQB1 | 7 | 18.92 | 31.27 | 6.45E-08 | 6.46E-06 |
| Arthritis, Rheumatoid\| | SLC22A4, IL1RL1, KIAA1109, CD247, HLA-DQA2, HLA-DQA1, HLA-DQB1 | 7 | 18.92 | 19.79 | 9.79E-07 | 8.41E-05 |
| Crohn Disease\|Crohn's disease | GSDMB, SLC22A5, SMAD3, ORMDL3, BACH2, HLA-DQA1 | 6 | 16.22 | 23.12 | 4.63E-06 | 3.48E-04 |
| diabetes, type 1 | SLC22A4, SLC22A5, IRF1, CLEC16A, PDCD1, HLA-DQA1, HLA-DQB1 | 7 | 18.92 | 14.48 | 6.02E-06 | 3.62E-04 |
| rheumatoid arthritis | SLC22A4, SLC22A5, CLEC16A, PDCD1, HLA-DQB2, HLA-DQA1, HLA-DQB1 | 7 | 18.92 | 14.48 | 6.02E-06 | 3.62E-04 |
| Bronchiolitis, Viral\|Respiratory Syncytial Virus Infections | SLC22A4, SLC22A5, IRF1, STAT6, GATA3, IL18R1, MUC5AC | 7 | 18.92 | 13.04 | 1.10E-05 | 5.50E-04 |
| Cholangitis, Sclerosing | SLC22A4, SLC22A5, HLA-DQA1, HLA-DQB1 | 4 | 10.81 | 68.36 | 2.34E-05 | 0.00108 |
| Crohn's disease | SLC22A4, SLC22A5, IRF1, ORMDL3, HLA-DQB1 | 5 | 13.51 | 25.52 | 3.64E-05 | 0.001562 |
| hepatitis C | IL1RL1, IRF1, IL18R1, HLA-DQA1, HLA-DQB1 | 5 | 13.51 | 23.68 | 4.89E-05 | 0.001959 |
| Asthma\|Myocardial Infarction | IL33, IL1RL1, WDR36 | 3 | 8.11 | 235.84 | 5.87E-05 | 0.002205 |
| Asthma\|Bronchial Hyperreactivity\|Hypersensitivity, Immediate | FLG, IL1RL1, IRF1, STAT6, GATA3 | 5 | 13.51 | 21.60 | 7.01E-05 | 0.00248 |
| Crohn Disease | SLC22A4, SLC22A5, SMAD3, BACH2, HLA-DQB1 | 5 | 13.51 | 20.69 | 8.30E-05 | 0.002676 |
| Graves' disease | IRF1, PDCD1, HLA-DQA1, HLA-DQB1 | 4 | 10.81 | 44.92 | 8.46E-05 | 0.002676 |
| diabetes, type 1 | SMAD3, CLEC16A, SUOX, BACH2, HLA-DQA1, HLA-DQB1 | 6 | 16.22 | 12.48 | 9.11E-05 | 0.002738 |
| Tuberculosis | SLC22A4, SLC22A5, IRF1, HLA-DQA1, HLA-DQB1 | 5 | 13.51 | 19.65 | 1.01E-04 | 0.0029 |
| plasma eosinophil count | IL1RL1, WDR36, TSLP | 3 | 8.11 | 168.45 | 1.23E-04 | 0.003337 |
| Lupus Erythematosus, Systemic | CLEC16A, CD247, PDCD1, HLA-DQA2, HLA-DQB2, HLA-DQA1, HLA-DQB1 | 7 | 18.92 | 8.41 | 1.28E-04 | 0.003337 |
| Asthma\| | IL1RL1, ORMDL3, IL18R1, HLA-DQA1, HLA-DQB1 | 5 | 13.51 | 16.66 | 1.92E-04 | 0.004813 |

**Supplementary Table S4. Frequency of genes in GAD Terms.** A total of 24 GAD terms were under 5% FDR. The frequency of genes were counted and a percentage calculated.

| **GENE** | **Number of Terms (total 23)** | **%** |
| --- | --- | --- |
| HLA-DQB1 | 17 | 73.91 |
| HLA-DQA1 | 16 | 69.57 |
| SLC22A5 | 10 | 43.48 |
| IL1RL1 | 10 | 43.48 |
| IRF1 | 9 | 39.13 |
| SLC22A4 | 9 | 39.13 |
| ORMDL3 | 6 | 26.09 |
| IL18R1 | 6 | 26.09 |
| CLEC16A | 6 | 26.09 |
| GSDMB | 5 | 21.74 |
| PDCD1 | 5 | 21.74 |
| BACH2 | 5 | 21.74 |
| IL33 | 4 | 17.39 |
| SMAD3 | 4 | 17.39 |
| STAT6 | 4 | 17.39 |
| TSLP | 3 | 13.04 |
| GATA3 | 3 | 13.04 |
| SUOX | 3 | 13.04 |
| KIAA1109 | 3 | 13.04 |
| CD247 | 3 | 13.04 |
| WDR36 | 3 | 13.04 |
| FLG | 2 | 8.70 |
| MUC5AC | 2 | 8.70 |
| HLA-DQA2 | 2 | 8.70 |
| HLA-DQB2 | 2 | 8.70 |

**Supplementary Table S5. DAVID Functional Annotation Tool analysis of GO Terms.** Candidate causal gene clusters and GO Term associations are listed. FE = fold enrichment.

| **Term** | **Genes** | **Count** | **%** | **FE** | **p-value** | **FDR** |
| --- | --- | --- | --- | --- | --- | --- |
| T cell costimulation | CD247, PDCD1, HLA-DQA2, HLA-DQB2, HLA-DQA1, HLA-DQB1 | 6 | 16.22 | 36.901 | 4.76E-07 | 1.50E-04 |
| MHC class II receptor activity | HLA-DQA2, HLA-DQB2, HLA-DQA1, HLA-DQB1 | 4 | 10.81 | 136.41 | 2.77E-06 | 2.86E-04 |
| MHC class II protein complex | HLA-DQA2, HLA-DQB2, HLA-DQA1, HLA-DQB1 | 4 | 10.81 | 92.04 | 9.75E-06 | 5.75E-04 |
| integral component of lumenal side of endoplasmic reticulum membrane | HLA-DQA2, HLA-DQB2, HLA-DQA1, HLA-DQB1 | 4 | 10.81 | 69.824 | 2.29E-05 | 6.76E-04 |
| antigen processing and presentation of peptide or polysaccharide antigen via MHC class II | HLA-DQA2, HLA-DQB2, HLA-DQA1, HLA-DQB1 | 4 | 10.81 | 112.89 | 5.06E-06 | 7.99E-04 |
| transport vesicle membrane | HLA-DQA2, HLA-DQB2, HLA-DQA1, HLA-DQB1 | 4 | 10.81 | 53.29 | 5.23E-05 | 9.71E-04 |
| clathrin-coated endocytic vesicle membrane | HLA-DQA2, HLA-DQB2, HLA-DQA1, HLA-DQB1 | 4 | 10.81 | 49.39 | 6.58E-05 | 9.71E-04 |
| T cell receptor signaling pathway | GATA3, CD247, HLA-DQA2, HLA-DQB2, HLA-DQA1, HLA-DQB1 | 6 | 16.22 | 19.45 | 1.13E-05 | 9.77E-04 |
| interferon-gamma-mediated signaling pathway | IRF1, HLA-DQA2, HLA-DQB2, HLA-DQA1, HLA-DQB1 | 5 | 13.51 | 33.79 | 1.24E-05 | 9.77E-04 |
| ER to Golgi transport vesicle membrane | HLA-DQA2, HLA-DQB2, HLA-DQA1, HLA-DQB1 | 4 | 10.81 | 38.94 | 1.34E-04 | 0.001587 |
| endocytic vesicle membrane | HLA-DQA2, HLA-DQB2, HLA-DQA1, HLA-DQB1 | 4 | 10.81 | 30.68 | 2.73E-04 | 0.002688 |
| trans-Golgi network membrane | HLA-DQA2, HLA-DQB2, HLA-DQA1, HLA-DQB1 | 4 | 10.81 | 24.40 | 5.37E-04 | 0.004523 |

**Supplementary Table S6. DAVID Functional Annotation Tool analysis of the KEGG/REACTOME Pathway.** Candidate causal gene clusters and pathway associations are listed from the GAD database. FE = fold enrichment.

| **Category** | **Term** | **Genes** | **Count** | **%** | **P-value** | **FE** | **FDR** |
| --- | --- | --- | --- | --- | --- | --- | --- |
| KEGG | Inflammatory bowel disease | SMAD3, STAT6, RORA, HLA-DQA2, IL18R1, HLA-DQA1, HLA-DQB1 | 7 | 18.92 | 5.83E-09 | 41.80 | 2.86E-07 |
| REACTOME | PD-1 signalling | CD247, PDCD1, HLA-DQA2, HLA-DQB2, HLA-DQA1, HLA-DQB1 | 6 | 16.22 | 4.17E-09 | 87.26 | 2.92E-07 |
| REACTOME | Translocation of ZAP-70 to Immunological synapse | CD247, HLA-DQA2, HLA-DQB2, HLA-DQA1, HLA-DQB1 | 5 | 13.51 | 2.23E-07 | 85.94 | 7.79E-06 |
| REACTOME | Phosphorylation of CD3 and TCR zeta chains | CD247, HLA-DQA2, HLA-DQB2, HLA-DQA1, HLA-DQB1 | 5 | 13.51 | 3.83E-07 | 75.63 | 8.93E-06 |
| REACTOME | Generation of second messenger molecules | CD247, HLA-DQA2, HLA-DQB2, HLA-DQA1, HLA-DQB1 | 5 | 13.51 | 1.75E-06 | 52.52 | 3.06E-05 |
| REACTOME | Interferon gamma signaling | IRF1, HLA-DQA2, HLA-DQB2, HLA-DQA1, HLA-DQB1 | 5 | 13.51 | 6.35E-05 | 21.48 | 8.89E-04 |
| REACTOME | Downstream TCR signaling | CD247, HLA-DQA2, HLA-DQB2, HLA-DQA1, HLA-DQB1 | 5 | 13.51 | 1.09E-04 | 18.72 | 0.001269 |
| REACTOME | MHC class II antigen presentation | HLA-DQA2, HLA-DQB2, HLA-DQA1, HLA-DQB1 | 4 | 10.81 | 0.003449 | 12.40 | 0.034495 |
| KEGG | Asthma | HLA-DQA2, HLA-DQA1, HLA-DQB1 | 3 | 8.11 | 0.002401 | 38.22 | 0.037734 |
| KEGG | Graft-versus-host disease | HLA-DQA2, HLA-DQA1, HLA-DQB1 | 3 | 8.11 | 0.002902 | 34.74 | 0.037734 |
| KEGG | Allograft rejection | HLA-DQA2, HLA-DQA1, HLA-DQB1 | 3 | 8.11 | 0.003639 | 30.99 | 0.037734 |
| KEGG | Type I diabetes mellitus | HLA-DQA2, HLA-DQA1, HLA-DQB1 | 3 | 8.11 | 0.00467 | 27.30 | 0.037734 |
| KEGG | Cell adhesion molecules (CAMs) | PDCD1, HLA-DQA2, HLA-DQA1, HLA-DQB1 | 4 | 10.81 | 0.004736 | 10.77 | 0.037734 |
| KEGG | Intestinal immune network for IgA production | HLA-DQA2, HLA-DQA1, HLA-DQB1 | 3 | 8.11 | 0.005821 | 24.39 | 0.037734 |
| KEGG | Autoimmune thyroid disease | HLA-DQA2, HLA-DQA1, HLA-DQB1 | 3 | 8.11 | 0.007089 | 22.05 | 0.037734 |
| KEGG | Staphylococcus aureus infection | HLA-DQA2, HLA-DQA1, HLA-DQB1 | 3 | 8.11 | 0.007628 | 21.23 | 0.037734 |
| KEGG | Influenza A | IL33, HLA-DQA2, HLA-DQA1, HLA-DQB1 | 4 | 10.81 | 0.00833 | 8.79 | 0.037734 |
| KEGG | Viral myocarditis | HLA-DQA2, HLA-DQA1, HLA-DQB1 | 3 | 8.11 | 0.008471 | 20.11 | 0.037734 |

**Supplementary Table S7. GeneMANIA scores for predicted genes.** The GeneMANIA tool was used to identify interactions between the 37 candidate genes and predict additional genes. Predicted gene scores were multiplied by the number of genes the predicted gene interacted with to obtain a total score and identify the predicted genes with the strongest interactions.

| **Predicted gene** | **score** | **No. of gene interactions** | **Total score** |
| --- | --- | --- | --- |
| CD274 | 6.06 | 13 | 78.78 |
| ZAP70 | 4.03 | 9 | 36.27 |
| PDCD1LG2 | 3.56 | 6 | 21.36 |
| IRAK4 | 2.99 | 5 | 14.95 |
| IL37 | 2.03 | 6 | 12.18 |
| SOX4 | 2.82 | 3 | 8.46 |
| IL18 | 2.04 | 4 | 8.16 |
| CRLF2 | 3.84 | 2 | 7.68 |
| CADPS | 2.41 | 3 | 7.23 |
| IL1RAP | 2.27 | 3 | 6.81 |
| TMED1 | 2.19 | 3 | 6.57 |
| PRL | 1.64 | 4 | 6.56 |
| TMBIM6 | 2.18 | 3 | 6.54 |
| TSR2 | 2.89 | 2 | 5.78 |
| KCNJ4 | 1.87 | 3 | 5.61 |
| SMAD4 | 2.37 | 2 | 4.74 |
| PCSK6 | 2.03 | 2 | 4.06 |
| ATXN1 | 2.63 | 1 | 2.63 |
| MAGED1 | 2.32 | 1 | 2.32 |
| CASP14 | 2.26 | 1 | 2.26 |


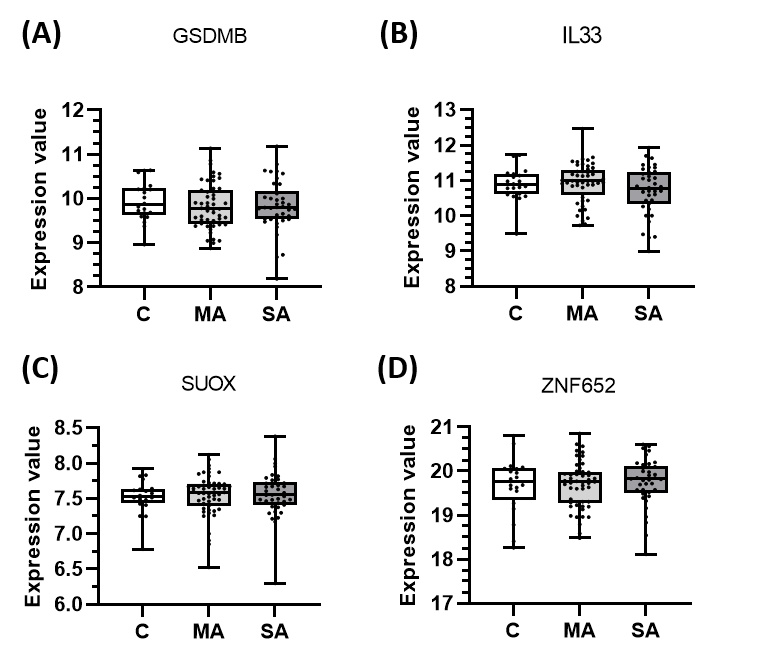


**Supplementary Figure S1. mRNA expression of candidate causal genes in bronchial epithelial cells taken from patients with asthma and controls.** Boxes shows the median and IQR and the whiskers show the minimum and maximum data. Bronchial epithelial brush samples were from controls (C, n=20) and patients with mild-moderate (MA, n=50) and severe (SA, n=38) asthma. Data is shown for **(A)** GSDMB, **(B)** IL33, **(C)** SUOX and **(D)** ZNF652. Expression values were taken from the dataset and a Kruskal-Wallis test with a two-stage linear step-up procedure of Benjamini, Krieger and Yekutieli used to control the FDR at 5% was performed.


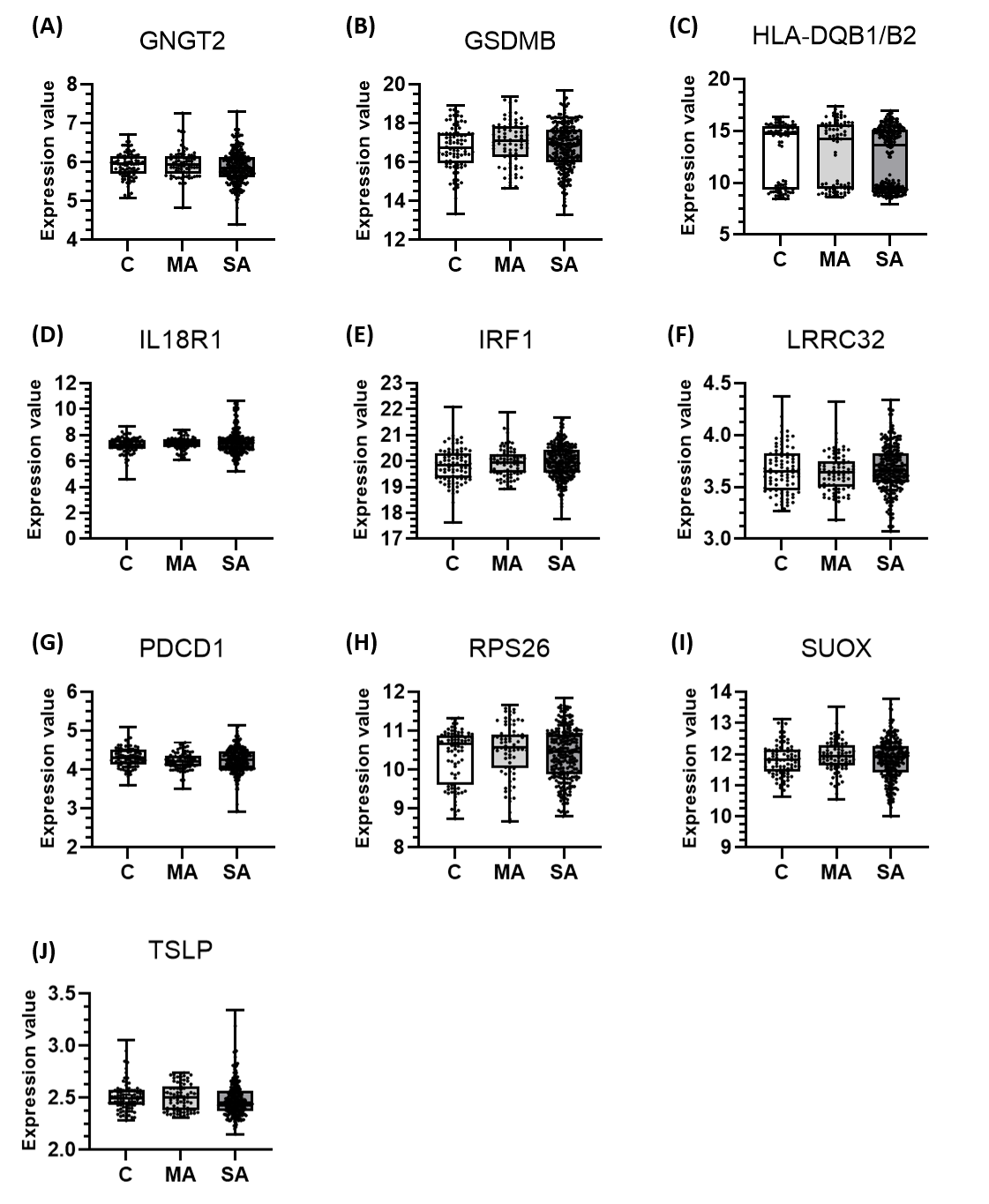


**Supplementary Figure S2.** **mRNA expression of candidate causal genes in blood taken from patients with asthma and control subjects.** Boxes shows the median and IQR and the whiskers show the minimum and maximum data. Blood samples were from controls (C, n=87) and patients with mild-moderate (MA, n=77) and severe (SA, n=246) asthma. Data is shown for **(A)** GNGT2, **(B)** GSDMB, **(C)** HLA-DQB1/B2, **(D)** IL18R1, **(E)** IRF1, **(F)** LRRC32, **(G)** PDCD1, **(H)** RPS26, **(I)** SUOX and **(J)** TSLP. Expression values were taken from the dataset and either a Kruskal-Wallis test or Welch’s ANOVA (GSDMB), both with a two-stage linear step-up procedure of Benjamini, Krieger and Yekutieli used to control the FDR at 5%, was performed.
